# Overexpression of Latent TGFβ Binding Protein 4 in Muscle Ameliorates Muscular Dystrophy through Myostatin and TGFβ

**DOI:** 10.1371/journal.pgen.1006019

**Published:** 2016-05-05

**Authors:** Kay-Marie Lamar, Sasha Bogdanovich, Brandon B. Gardner, Quan Q. Gao, Tamari Miller, Judy U. Earley, Michele Hadhazy, Andy H. Vo, Lisa Wren, Jeffery D. Molkentin, Elizabeth M. McNally

**Affiliations:** 1 Department of Human Genetics, The University of Chicago, Chicago, Illinois, United States of America; 2 Center for Genetic Medicine, Northwestern University Feinberg School of Medicine, Chicago Illinois, United States of America; 3 Molecular Pathogenesis and Molecular Medicine, The University of Chicago, Chicago, Illinois, United States of America; 4 Committee on Development, Regeneration, and Stem Cell Biology, The University of Chicago, Chicago, Illinois, United States of America; 5 Department of Medicine, The University of Chicago, Chicago, Illinois, United States of America; 6 Cincinnati Children’s Hospital, Howard Hughes Medicine Institutes, Cincinnati, Ohio, United States of America; The Jackson Laboratory, UNITED STATES

## Abstract

Latent TGFβ binding proteins (LTBPs) regulate the extracellular availability of latent TGFβ. LTBP4 was identified as a genetic modifier of muscular dystrophy in mice and humans. An in-frame insertion polymorphism in the murine *Ltbp4* gene associates with partial protection against muscular dystrophy. In humans, nonsynonymous single nucleotide polymorphisms in *LTBP4* associate with prolonged ambulation in Duchenne muscular dystrophy. To better understand LTBP4 and its role in modifying muscular dystrophy, we created transgenic mice overexpressing the protective murine allele of *LTBP4* specifically in mature myofibers using the human skeletal actin promoter. Overexpression of LTBP4 protein was associated with increased muscle mass and proportionally increased strength compared to age-matched controls. In order to assess the effects of LTBP4 in muscular dystrophy, LTBP4 overexpressing mice were bred to *mdx* mice, a model of Duchenne muscular dystrophy. In this model, increased LTBP4 led to greater muscle mass with proportionally increased strength, and decreased fibrosis. The increase in muscle mass and reduction in fibrosis were similar to what occurs when myostatin, a related TGFβ family member and negative regulator of muscle mass, was deleted in *mdx* mice. Supporting this, we found that myostatin forms a complex with LTBP4 and that overexpression of LTBP4 led to a decrease in myostatin levels. LTBP4 also interacted with TGFβ and GDF11, a protein highly related to myostatin. These data identify LTBP4 as a multi-TGFβ family ligand binding protein with the capacity to modify muscle disease through overexpression.

## Author Summary

Muscular dystrophy is a genetic disease with muscle weakness, replacement of muscle tissue with fibrosis, and premature death. The gene for latent TGFβ binding protein 4 (*LTBP4*) was previously found to modify muscular dystrophy in both mice and humans with variants that confer protection from disease. In order to better understand this modifier gene, the protective version of LTBP4 was overexpressed specifically in the skeletal muscles of mice. Increased levels of LTBP4 protein resulted in increased muscle mass. Overexpression of LTBP4 in a mouse model of Duchenne muscular dystrophy alleviated many disease-associated features producing larger muscles, increased strength, and reduced fibrosis in muscle. LTBP4 formed a complex with myostatin, a protein that when inhibited leads to muscle growth. In LTBP4-overexpressing mice, active myostatin protein was decreased. This study shows that LTBP4 modifies muscular dystrophy based on its ability to scaffold and regulate multiple TGFβ family members including myostatin.

## Introduction

Latent TGFβ binding proteins (LTBPs) are extracellular matrix (ECM) proteins that bind and sequester the small latent complex of TGFβ. There are more than 30 TGFβ family members, and the primary TGFβ family members 1, 2 and 3 are known to coordinate cell growth during development and regulate response to injury in mature cell and tissue types (reviewed in [1–4]). Other members of the TGFβ family include the inhibins/activins, bone morphogenetic proteins (BMPs) and the growth and differentiation factors (GDFs). Two GDFs are especially important for muscle health. Deletion of GDF8, known as myostatin, leads to doubling of muscle mass in mice, and naturally occurring mutations in the *MSTN* gene result in increased muscle mass in large animals and humans [5–8]. GDF11 is nearly identical to GDF8/myostatin in its active domain, and, although controversial, GDF11 has been linked to muscle wasting in aging [9, 10].

TGFβ family members reside in the extracellular matrix, where their activity is regulated through sequestration by latency complexes (reviewed in [11, 12]). By binding to matrix components, the activity of TGFβ proteins is tightly controlled with multiple levels of inhibition. The active domain of TGFβ first forms an inactive complex by binding its prodomain, referred to as the latency associated peptide or LAP. Together TGFβ and LAP form the small latent complex. The small latent complex is found associated with LTBP in the matrix, as the large latent complex (reviewed in [11]). In the extracellular matrix, active TGFβ proteins are liberated from LTBPs by proteolytic or force-induced conformational change and engage the TGFβ receptor only after release of both LTBP and LAP [13–15].

Four LTBPs (1 to 4) share structural similarity but display distinct expression patterns [16]. LTBP4 is expressed highly in the heart, skeletal muscle, and smooth muscle and is expressed at lower levels in other tissues [16, 17]. In humans, multiple LTBP4 forms are present, and two of these that differ at the amino terminus were characterized as being transcribed from two separate promoters [18]. The long isoform (LTBP-4L) is thought to have a higher affinity for TGFβ1 compared to the short isoform (LTBP-4S) [18]. Mice deficient in the short isoform of *Ltbp4* display a syndrome of pulmonary emphysema, colorectal cancer, and cardiomyopathy [19]. In mice, a genetic deletion that targets both *Ltbp4* isoforms produces a more severe neonatal lethal phenotype including abnormalities of the skin, lung, and aorta [20]. Humans with recessive *LTBP4* loss of function mutations have a multi-organ syndrome with impaired pulmonary, gastrointestinal, genitourinary, musculoskeletal, and dermal development [21]. These findings underscore the importance of regulating TGFβ during development.

A genomewide quantitative trait locus (QTL) screen in mice identified *Ltbp4* as a genetic modifier of muscular dystrophy [22]. In mice, there are two alleles of *Ltbp4* that differ at an insertion/deletion polymorphism that alters the hinge region of the protein. The majority of mouse strains carry the insertion *Ltbp4* allele. In the setting of muscular dystrophy, the protective *Ltbp4* allele was associated with increased grip strength, improved muscle membrane leak, and reduced fibrosis in the γ-sarcoglycan null (*Sgcg*) model of muscular dystrophy [22]. Complementing the studies in mice, non-synonymous single nucleotide polymorphisms (SNPs) in *LTBP4* were associated with age at loss of ambulation in human Duchenne muscular dystrophy [23–25].

To assess the mechanisms by which LTBP4 acts in skeletal muscle and in muscular dystrophy, we generated transgenic mice overexpressing the protective allele of murine *Ltbp4* using a promoter driving expression exclusively in skeletal muscle. Transgenic positive (TG+) mice expressed elevated levels of LTBP4 protein in muscle, and exhibited increased muscle mass and proportionally increased strength. When this protective *Ltbp4* allele was introduced into the *mdx* mouse model for Duchenne Muscular Dystrophy, these mice had significantly reduced fibrosis and increased strength. We also identified that LTBP4 bound TGFβ 1, 2 and 3, and found that LTBP4 bound myostatin and the highly related GDF11. Active levels of myostatin were reduced in transgenic *mdx* muscle, providing a mechanism by which this protein exerts its protective effects in muscular dystrophy.

## Results

### Overexpression of *LTBP4* promotes increased muscle mass and strength

**Fig 1:**
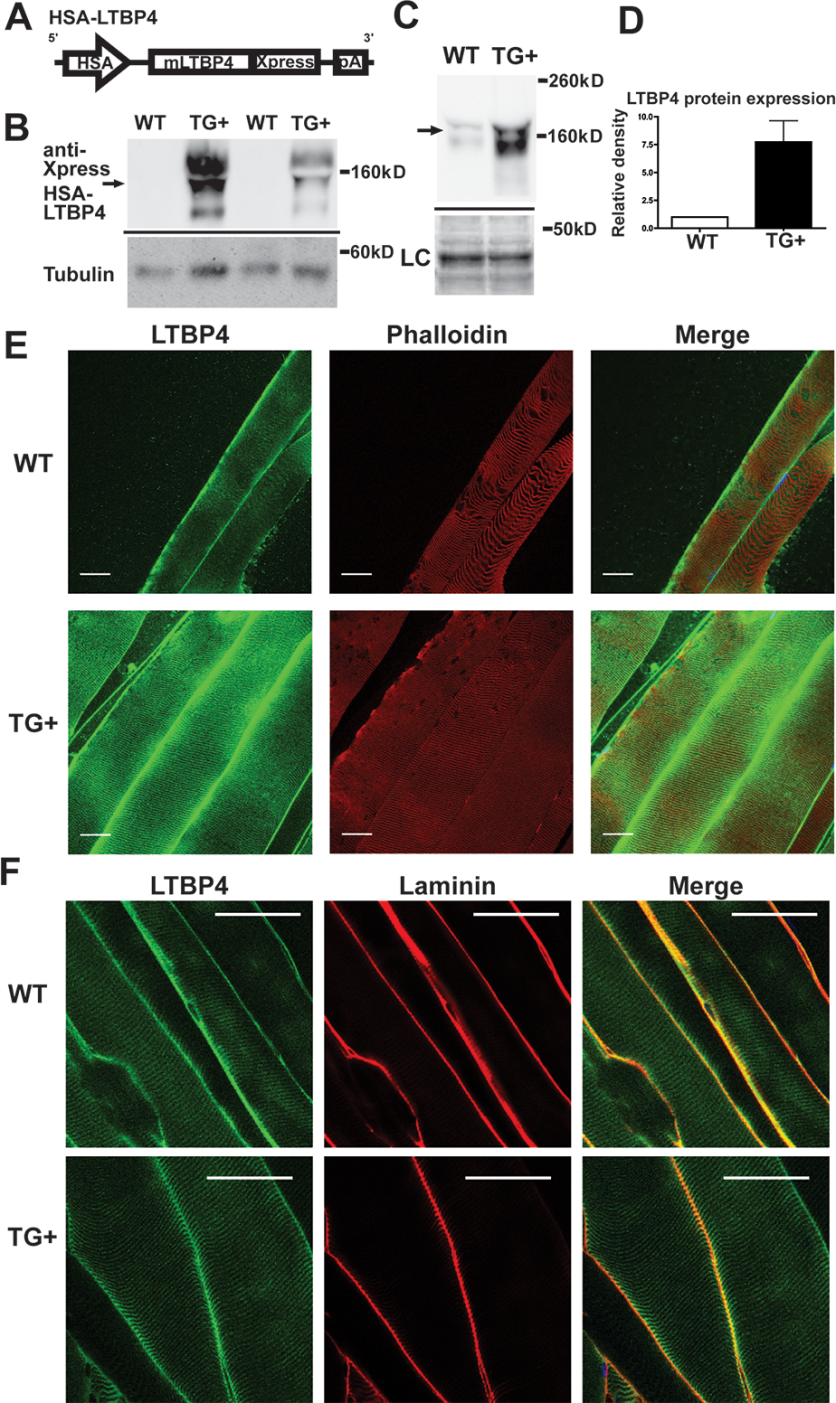
Transgenic overexpression of LTBP4 in muscle. **(A)** Mouse *LTBP4* (mLTBP4) was overexpressed in skeletal muscle under control of the human skeletal actin (HSA) promoter. **(B)** Quadriceps muscle lysates from wildtype and TG+ mice were immunoblotted for the anti-Xpress tag on the *LTBP4* transgene demonstrating LTBP4 protein overexpression. **(C)** Quadriceps muscle lysates similarly immunoblotted using an antibody to LTBP4 to assess overall LTBP4 expression in muscle (arrow). LC, loading control, represent the MemCode staining for actin. **(D)** Quantification of immunoblots from wildtype (n = 2) and TG+ (n = 3) muscle lysates showed that the *LTBP4* TG+ mice express ~7 times the amount of LTBP4 protein of wildtype mice. **(E)** Individual myofibers were isolated from TG+ and wildtype control mice and incubated with antibodies to LTBP4 under conditions that did not allow antibody permeabilization. LTBP4 localizes sarcolemma in costameric pattern and extracellular matrix in murine muscle, and TG+ mice have qualitatively more LTBP4 protein expression. Scale bar = 30 μm. **(F)** Co-immunostaining with an anti-laminin antibody (red) demonstrates the extracellular location of LTBP4 (green) and shown in merged image as yellow. Scale bar 50 μm.

*LTBP4* was previously shown to modify muscular dystrophy in humans and mice [22, 23]. In order to better assess the mechanism underlying this modifying effect, we first characterized the effect of transgenic overexpression of LTBP4 in wildtype skeletal muscle. Transgenic mice were generated overexpressing the protective allele of murine *Ltbp4* under control of the human skeletal α-actin (HSA) promoter and the troponin-I slow upstream enhancer (HSA-LTBP4) (Fig 1A) [26, 27]. The HSA promoter coupled with the troponin enhancer drives high-level expression in postnatal skeletal muscle, specifically myosin heavy chain type IIB fibers [28]. The *Ltbp4* transgene was engineered with an Xpress epitope tag on its carboxy- terminus to permit specific detection of the transgene (Fig 1A). Three independent lines were generated with a range of expression (lines 5, 6, and 11). We selected line 6 for further characterization since it displayed an intermediate level of expression between the other two. The transgene from line 6 randomly inserted into the Y chromosome; therefore male mice were used for all comparisons. Immunoblotting quadriceps muscle lysates for the anti-Xpress epitope tag confirmed protein expression from the transgene (Fig 1B). Using an antibody to LTBP4, the relative protein expression level of LTBP4 was measured from immunoblots of wildtype (n = 2) and TG+ (n = 3) mice quadriceps muscles (representative blot in Fig 1C). Quantification of immunoblots using an anti-LTBP4 antibody confirmed an approximately seven-fold increase in LTBP4 protein level in TG+ muscles compared to controls (Fig 1D). The HSA promoter was not observed to drive appreciable expression in the diaphragm muscle, even in the high expressing line, consistent with the known activity of this promoter (S1 Fig) [28].

Confocal immunofluorescence microscopy was used to image LTBP4 protein expression in muscle fibers. Fibers were rapidly isolated and teased to visualize individual or small groups of fibers. Because LTBPs, including LTBP4, are extracellular proteins [29], immunostaining was performed in the absence of detergent or other permeabilizing agent so that antibodies did not gain entry into myofibers and to visualize the extracellular pattern of LTBP4 [30]. LTBP4 immunoreactivity was found enriched along the surface of myofibers in a striated pattern, consistent with costameric positioning along the sarcolemma (Fig 1E). The pattern seen with phalloidin, shown in red, to represent intracellular actin, also showed the expected striated sarcomeric pattern. However, there was no merged signal in yellow, indicating LTBP4 and actin are found in a parallel but nonoverlapping pattern. Wildtype control fibers showed similar localization of LTBP4 protein, but the TG+ muscles showed qualitatively more LTBP4 staining in both the membrane-associated and extracellular pools of LTBP4 (compare Fig 1E images, processed similarly and taken at identical exposures). To confirm the extracellular nature of LTBP4, co-localization with laminin was observed in the enrichment of LTBP4 seen between fibers, but not in the costameric pattern, representing the pool of LTBP4 found closely apposed to the sarcolemma (Fig 1F). Similarly, LTBP4 showed a similar pattern of co-localization with wheat germ agglutinin (WGA), a lectin that binds the matrix (S2 Fig). Similar to what we previously reported for LTBP4, it is found in two pools, one pools closely linked to the sarcolemmal surface and a second pool enriched in between fibers aligned with other matrix proteins [30].

**Fig 2:**
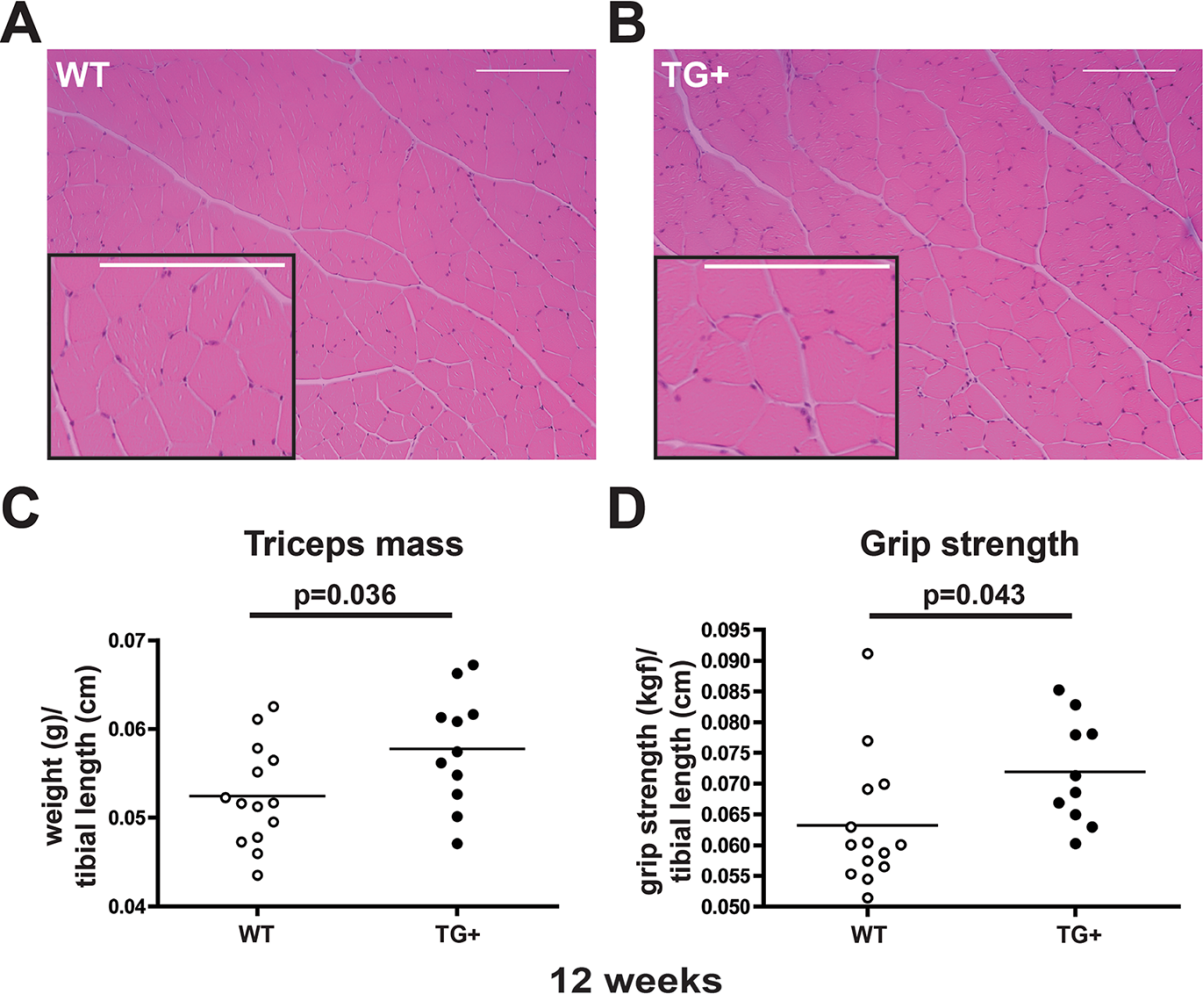
Larger muscles and increased grip strength in LTBP4 Tg+ mice. **(A,B)** Wildtype **(A)** and TG+ **(B)** quadriceps muscle sections were stained with hematoxylin and eosin at 12 weeks of age. TG+ muscle fibers have normal overall architecture without fibrosis or necrosis. **(C)** The mass of triceps muscles from TG+ mice (n = 11) was significantly greater mass than wildtype mice (n = 14) when normalized to tibial length. **(D)** TG+ mice (n = 10) have significantly increased forelimb grip strength compared to wildtype mice (n = 14). kGF, kilogram force.

Hematoxylin and eosin staining of transgenic positive muscle showed no evidence of fibrosis or necrotic fibers, which would be indicative of myopathy (Fig 2A and 2B). The mean fiber size in quadriceps muscle was comparable between WT and LTBP4 TG+ muscle, however LTBP4 TG+ muscle had an increase in the largest myofibers, especially those greater than 6500 μm2 (S3 Fig). In order to assess the phenotype of muscle-specific overexpression of *Ltbp4*, transgenic mice were examined for muscle mass and grip strength. Transgenic mice had significantly larger triceps and gastrocnemius/soleus mass than age-matched controls at 8 weeks (S4 Fig). At 12 weeks, TG+ mice had larger triceps mass compared to age-matched controls (p = 0.0360) (Fig 2C). To test whether this increase in muscle mass altered strength in these mice, we measured forelimb grip strength at 8 and 12 weeks. There was no significant difference in strength among 8-week animals. However, 12-week-old TG+ animals were stronger than controls (p = 0.0434) (Fig 2D); the larger mass correlated with a proportional increase in strength.

### Dystrophic mice overexpressing *LTBP4* have increased body and skeletal muscle mass

**Fig 3:**
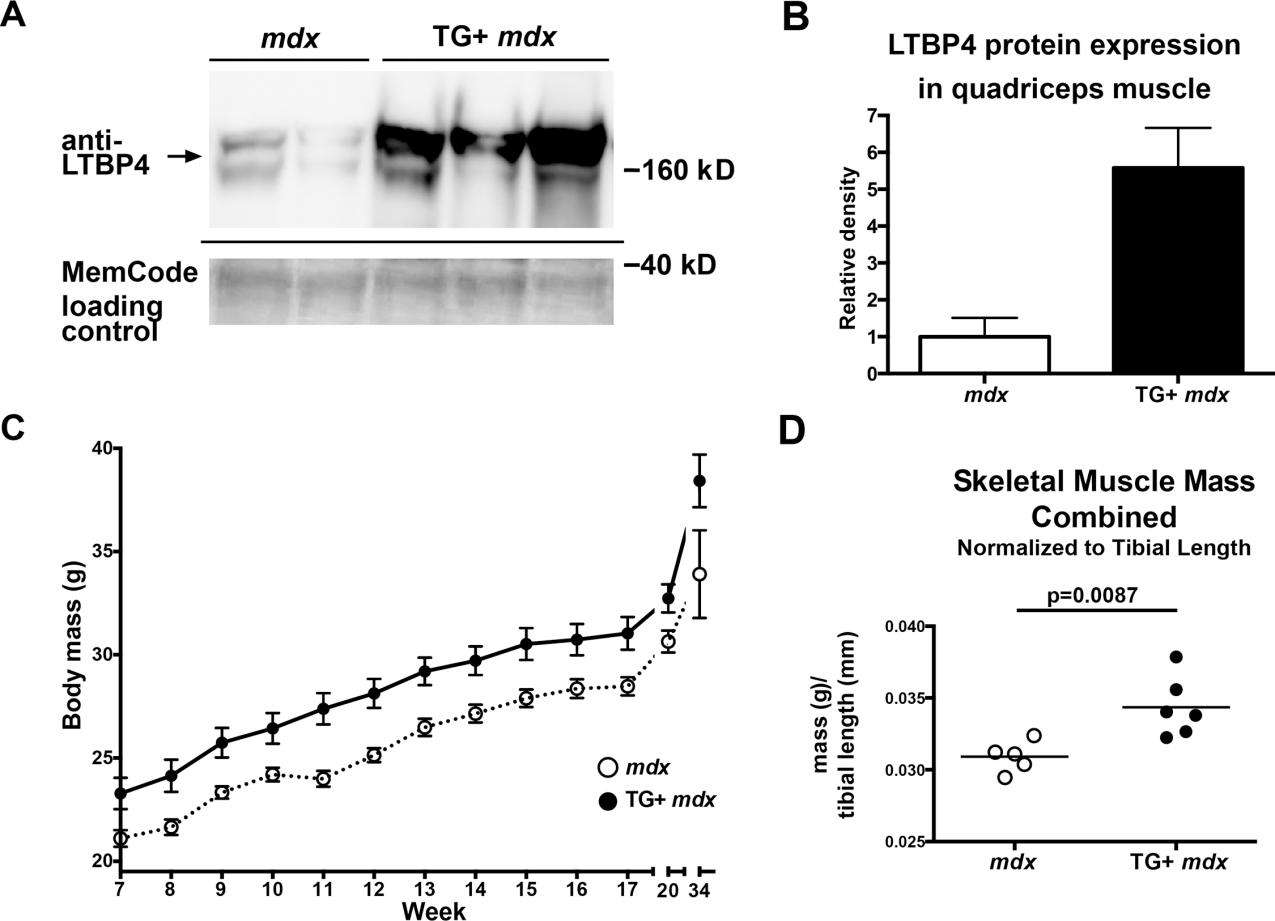
Increased body mass and skeletal muscle mass in LTBP4 TG+ *mdx* mice. LTBP4 TG+ mice were bred onto the *mdx-/-* background. **(A)** Quadriceps muscle lysates from TG+ *mdx* mice and *mdx* controls were immunoblotted using an antibody to LTBP4. **(B)** Quantification of LTBP4 expression from murine muscle lysates indicates that LTBP4 was expressed ~6 fold higher in TG+ *mdx* mice (n = 3) compared to *mdx* controls (n = 2). **(C)** Body mass was measured in TG+ *mdx* mice and *mdx* controls between the ages of 7 and 20 weeks (n = 13 each group) and also at 34 weeks (n = 5 for *mdx*, n = 6 for TG+*mdx*). TG+ *mdx* mice had significantly increased body weight (p<0.0001, 2-way ANOVA). **(D)** The masses of triceps, quadriceps, gluteus, hamstrings, gastrocnemius, and soleus muscles were obtained from 34 week old TG+ *mdx* mice (n = 6) and *mdx* controls (n = 5). The combined skeletal muscle mass of TG+ *mdx* mice was significantly increased compared to *mdx* mice (p = 0.0087, student’s t-test, two tailed).

We next evaluated the effect of LTBP4 skeletal muscle overexpression in the context of muscular dystrophy. LTBP4 overexpressing mice were bred with *mdx* mice, a mouse model of muscular dystrophy. LTBP4 protein levels were ~6 fold higher in quadriceps muscle from TG+ *mdx* mice compared to *mdx* controls (Fig 3A and 3B). TG+ *mdx* mice also had significantly larger body masses throughout their lives (7–34 weeks) compared to *mdx* controls (p<0.0001, two way ANOVA), (Fig 3C). To investigate whether this increase in body mass was associated with an increase in muscle mass, TG+ *mdx* muscle masses were measured at 34 weeks of age and compared to muscles masses of *mdx*. Because of the overall increase in body mass, muscle mass was normalized to tibial bone length, since long bone growth ceases with epiphyseal fusion after puberty [31]. Skeletal muscle mass was increased in TG+ *mdx* mice compared to *mdx* controls when normalized to tibial length (Fig 3D and S5 Fig). Interestingly, non-skeletal muscle tissues (e.g. heart, kidney) from TG+ *mdx* mice also showed significantly increased mass compared to *mdx*, indicating that upregulation of *Ltbp4* in muscle may exert a systemic effect (S5 Fig).

### LTBP4 associates with myostatin

**Fig 4:**
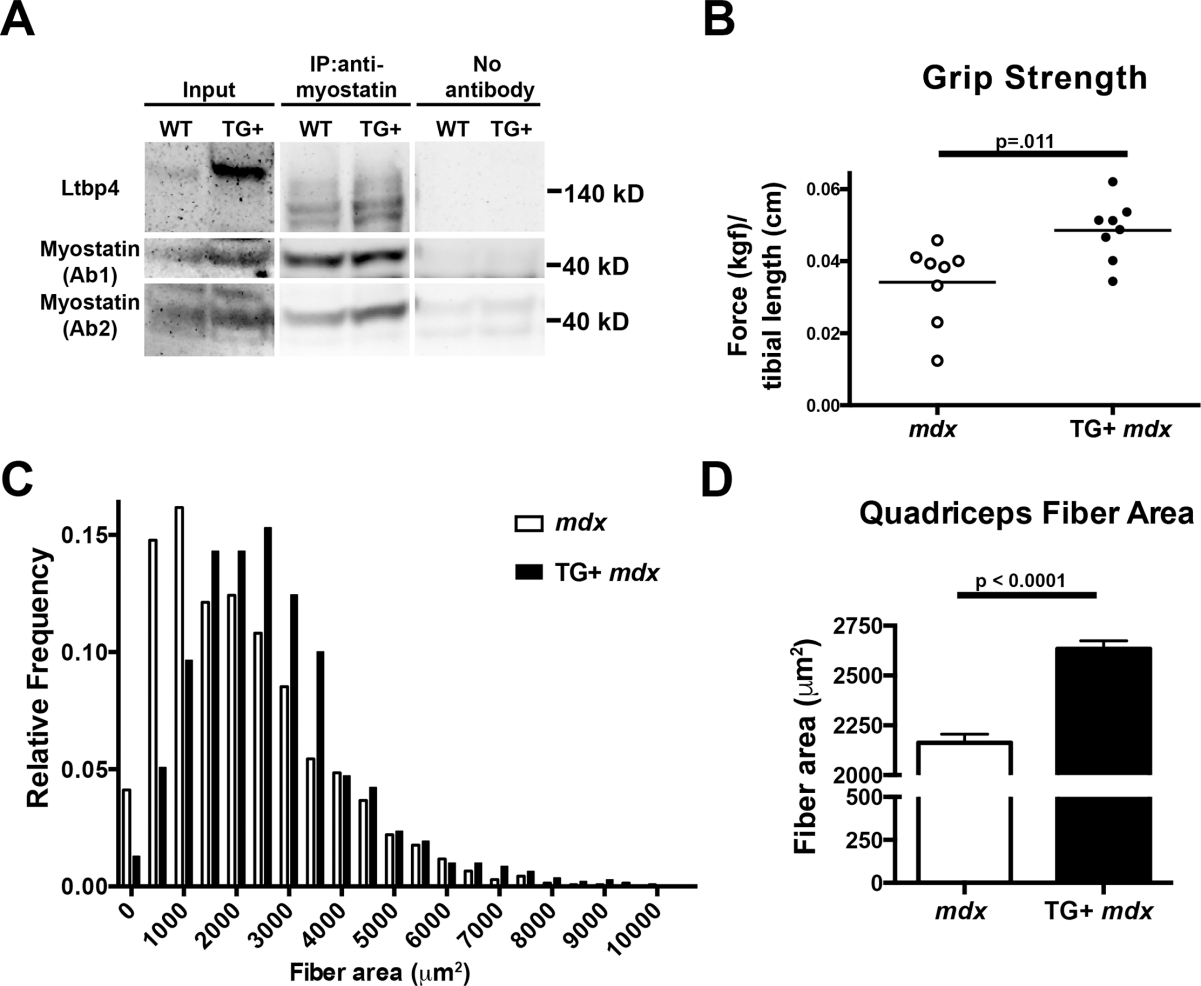
LTBP4 interacts with myostatin, and mice overexpressing LTBP4 have increased strength and bigger muscle fibers. **(A)** 10 week old TG+ and WT mice were sacrificed and muscle lysates were obtained by combining quadriceps, gastrocnemius, soleus, gluteus, hamstring, and triceps muscles. Lysates were immunoprecipitated with an anti-myostatin followed by immunoblotting with anti-LTBP4 antibody, showing that LTBP4 associates with myostatin in vivo. **(B)** Forelimb grip strength of TG+ *mdx* (n = 8) and *mdx* (n = 8) mice was measured over ten trials, averaged, and normalized to tibial length. (kgf, kilogram force) Twenty week old TG+ *mdx* mice were significantly stronger than *mdx* controls (p = .011, Student’s t-test). **(C)** Fiber area was determined by outlining hematoxylin and eosin stained quadriceps muscle fibers from TG+ *mdx* mice (n = 7) and *mdx* controls (n = 6) at 8 weeks of age. A histogram of fiber area shows that TG+ *mdx* mice had fewer small fibers and an increased number of large fibers. **(D)** TG+ *mdx* mice had significantly increased fiber area compared to *mdx* controls (p<0.0001, student’s t-test)

Myostatin is a member of the TGFβ family of proteins notable for its role as a negative regulator of muscle mass [5]. When the *Mstn* null allele was introduced into the *mdx* mice it resulted in increased mass and reduced fibrosis [32]. The results from *Ltbp4* overexpression in *mdx* mice were reminiscent to those reported for *Mstn* null/*mdx* mice prompting an evaluation of myostatin in *Ltbp4* transgenic mice. Immunoprecipitation was carried out on extracts from wildtype and TG+ muscle using an antibody to myostatin followed by immunoblotting with an anti-LTBP4 antibody. LTBP4 was present in the lanes where anti-myostatin antibody was used, whereas no LTBP4 was detected in the control lanes without immunoprecipitating antibody, consistent with an interaction between LTBP4 and myostatin (Fig 4A).

In order to test whether the increased muscle mass observed in TG+ *mdx* mice translated to functional improvement of the muscle, forelimb grip strength was measured in mice at 20 weeks. Forelimb grip strength measurements were significantly higher in TG+ *mdx* mice compared to *mdx* controls (p = 0.011) (Fig 4B). Furthermore, the distribution of fiber area from quadriceps muscles shows that TG+ *mdx* mice have fewer small fibers and an increased number of large fibers compared to *mdx* controls at 8 weeks of age (Fig 4C). Fiber area was quantified and we found TG+ *mdx* mice have significantly larger quadriceps muscle fibers compared to *mdx* controls, indicating that the increased muscle mass and strength in these mice can be attributed to muscle fiber hypertrophy (p<0.0001) (Fig 4D). The mean fiber area of wildtype animals from the C57Bl6/J strain is ~3100 μm^2^. Thus the increase in fiber area produced from the LTBP4 transgene in the mixed mdx C57Bl6/10 background does not exceed that of wildtype.

### Overexpression of *LTBP4* downregulates myostatin expression in skeletal muscle

**Fig 5:**
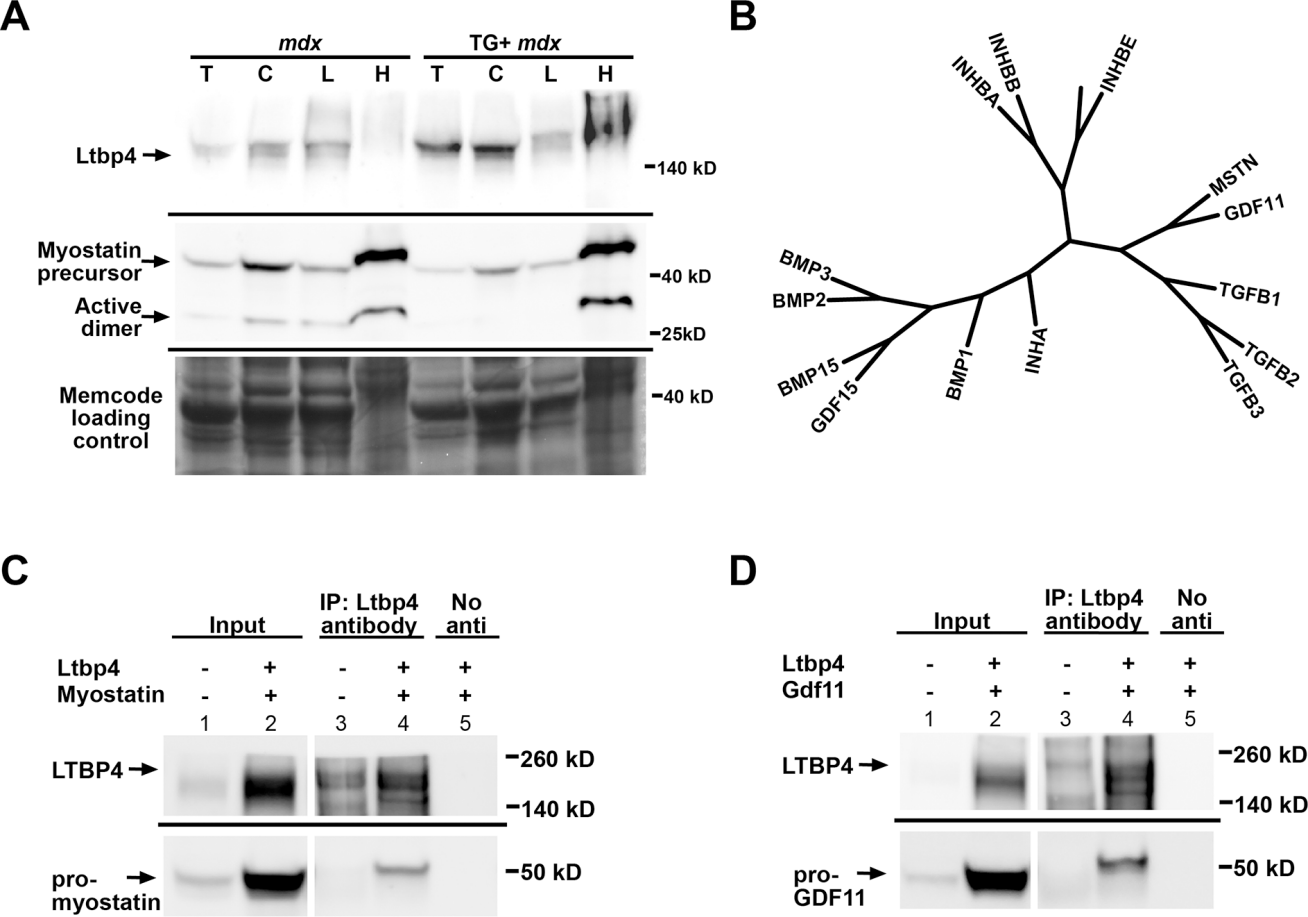
LTBP4 is a multi-cytokine regulator. **(A)** Muscle lysates were fractionated from total lysate (T) into cytosolic (C), light microsome (L) and heavy microsome (H) components. Individual fractions were immunoblotted with an antibody against the active domain of myostatin. There was less myostatin expressed in the soluble fractions of TG+ *mdx* mice muscle lysates compared to *mdx* controls. **(B)** A phylogenetic tree of mouse TGFβ family members derived from protein alignments illustrates the high similarity between these proteins, and explain why antibodies to myostatin cross react to GDF11. **(C, D)** Mouse LTBP4 and either myc-epitope-tagged myostatin **(C)** or myc-tagged GDF11 **(D)** were heterologous expressed in HEK293T cells. The myc-tagged myostatin and GDF11 were the complete prodomains and active domains together. Co-immunoprecipitation was performed on cell lysates by immunoprecipitating with anti-LTBP4 antibody followed by immunoblotting with anti-myc antibody detecting myostatin or GDF11. A co-IP control for each experiment was performed without adding IP antibody (labeled as “No anti”). LTBP4 was found to associate with both myostatin and GDF11 in vitro. The 49 KDa band in the first left lane of panels C and D likely represents endogenous myc.

LTBPs are covalently linked to the prodomains of latent TGFβ but are thought to form noncovalent links to other factors [33]. Because of an observed interaction between LTBP4 and myostatin, we evaluated whether overexpression of *LTBP4* had altered the amount of active myostatin in muscle. We performed cell fractionation on muscle lysates from *mdx* and TG+ *mdx* mice (n = 5) to obtain total, cytoplasmic, light microsomal, and heavy microsomal muscle fractions. The cytoplasmic and light microsomal fractions enrich for proteins that are more readily solubilized or less tightly linked to other proteins. The heavy microsomal fraction contains the less readily solubilized and typically membrane-associated components including the endoplasmic reticulum, Golgi, and plasma membrane. We immunoblotted fractionated lysates using an antibody to the carboxy terminus of myostatin, to track both the active and precursor forms of myostatin. We found that the soluble fractions from TG+ mdx muscle have significantly less myostatin expression than mdx muscle (Fig 5A and S6 Fig). Two bands were observed, one at ~26 kDa corresponding to the active myostatin dimer, and a ~42 kDa band corresponding to the myostatin precursor protein [13, 34, 35]. Active myostatin was expressed at a lower level in total lysates from TG+ *mdx* mice (S6 Fig, first lane T, n = 5 of each genotype). The level of the active myostatin was also reduced in the cytoplasmic and light microsomal fractions; the cytoplasmic fraction, which has a reduction to 18% of WT level (95% CI = (-0.2884–0.6570)), (Fig 5A and S6 Fig).

These results support a model where LTBP4 sequesters myostatin, in both its latent and active forms, in the extracellular matrix thereby inhibiting the release of active myostatin. The soluble fractions, which would represent the released component of myostatin, showed the greatest difference between *mdx* and TG+ *mdx* muscle. This release of active myostatin may reflect myostatin non-covalently linked to LTBP4 or methods that preferentially reduce covalently bonds between LTBP4 and myostatin in the fractionation procedure. This downregulation of myostatin in TG+ *mdx* mice provides support for the observed increase in muscle mass, similar to what is seen in *Mstn* null mice.

### LTBP4 is a multi-cytokine regulator

The TGFβ family includes subdomains of GDFs, BMPs, activins, as well as TGFβ isoforms 1–3. Multiple Sequence Alignment was conducted to depict the relationship among of a subgroup of human TGFβ family members (Fig 5B). Myostatin and GDF11 are the most closely related family members to TGFβ 1, 2 and 3. GDF11 shows increased expression in human muscles post-trauma and is 90% identical to myostatin in its mature region [36, 37]. Despite being highly related, myostatin and GDF11 have different expression patterns and distinct functions [5, 37]. In several recent papers GDF11 was suggested to be a rejuvenating factor for aged skeletal muscle, although this result has not been consistently observed [9, 10, 38].

Because myostatin and GDF11 are so similar, many antibodies to myostatin cross-react with GDF11 and vice versa [10, 39]. To circumvent this issue, we utilized a myc-tagged myostatin to confirm LTBP4’s interaction with myostatin *in vitro* (Fig 5C). HEK293T cells were transfected with constructs expressing mouse *Ltbp4* and myc-tagged myostatin. Co-immunoprecipitation with anti-LTBP4 antibodies, followed by immunoblotting with anti-myc antibody, demonstrated that myostatin formed a complex with LTBP4 (Fig 5C, lane 4). This interaction was dependent on the presence of LTBP4, as the control without anti-LTBP4 failed to show any interaction (Fig 5C, lane 5). Immunoprecipitation with an antibody against LTBP4 followed by immunoblotting with an anti-myc antibody detected GDF11, indicating that LTBP4 also binds GDF11 (Fig 5D, lane 4). Previous studies have found that levels of circulating myostatin are higher than those of GDF11, suggesting that GDF11 may be less physiologically significant than myostatin [39]. However, circulating myostatin levels may have less bearing on possible autocrine or paracrine roles of GDF11. Therefore, in order to assess the relative abundance of TGFβ isoforms, GDF11 and myostatin in muscle, RNA sequencing was performed on mouse skeletal muscles. RNA sequencing results show that the levels of myostatin transcript are approximately ten times higher than those of GDF11 in both abdominal and quadriceps muscles (Table 1). While mRNA levels may only partly correlate with protein levels, these results suggest that myostatin likely contributes more significantly to the myostatin/GDF11 pool within muscle. Therefore, we expect that it is myostatin sequestration that is most relevant to the findings from LTBP4 overexpression in muscle.

**Table 1:** RNA sequencing reveals relative abundance of TGFβ family members in muscle.

Gene	Gene Description	FPKM value Abdominal Muscle	FPKM value Quadriceps Muscle
*Tgfb1*	transforming growth factor, beta 1	2.6389	2.7175
*Tgfb2*	transforming growth factor, beta 2	7.4401	5.7572
*Tgfb3*	transforming growth factor, beta 3	19.0089	10.0939
*Gdf11*	growth differentiation factor 11	2.3267	2.1168
*Mstn*	myostatin	22.9699	21.0589

FPKM (fragment per kilobase of exon per million fragments mapped X10).

### Myostatin interacts with the amino terminus of LTBP4

**Fig 6:**
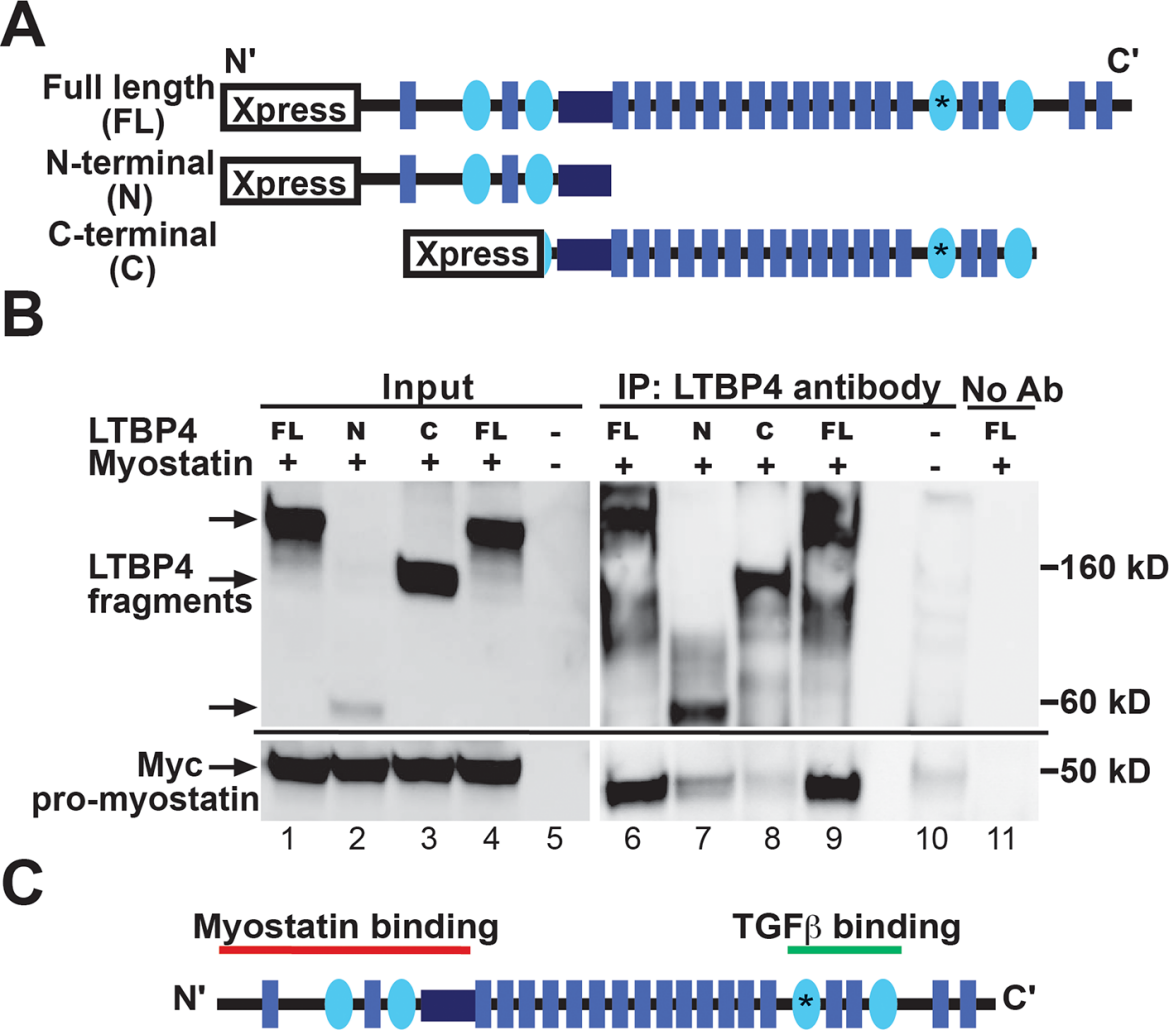
Myostatin binds the amino-terminus of LTBP4. **(A)** Full length (FL), amino terminal (N), and carboxy terminal (C) fragments of LTBP4 were ligated into expression vectors. **(B)** HEK293T cells were transfected with human LTBP4 and myostatin constructs. Co-immunoprecipitation was performed on cell lysates by immunoprecipitating with an anti-LTBP4 antibody followed by immunoblotting with anti-myc antibody detecting myostatin. A co-IP control was performed without adding IP antibody. Myostatin binds the amino terminus of LTBP4. **(C)** TGFβ is known to bind the carboxy-terminal region of LTBPs [40]. A schematic of LTBP4 protein shows the distinct binding sites of myostatin and TGFβ.

To refine the myostatin-LTBP4 interaction and to verify that human myostatin and LTBP4 interact, human LTBP4 and myostatin clones were transfected into HEK293T cells (Fig 6A). Co-immunoprecipitation with anti-LTBP4 antibodies, followed by immunoblotting with anti-myc antibody, demonstrated that human myostatin associates with human LTBP4 (Fig 6B, lanes 6 and 9). TGFβ1 binds LTBP proteins at the carboxy terminus at the third 8-cysteine-containing repeat [40]. To assess the region of LTBP4 responsible for the myostatin interaction, HEK293T cells were co-transfected with myostatin and constructs expressing either the amino or carboxy regions of LTBP4 (Fig 6A). Cell lysates were immunoprecipitated using an antibody to LTBP4 and immunoblotted using an antibody to the myc tag on myostatin (Fig 6B). Myostatin interacted with the amino terminus of LTBP4 and not the carboxy terminus (compare lane 7 to lane 8 in Fig 6B, bottom panel). These results indicate that myostatin interacts with a distinct site from the described TGFβ-binding domain on LTBP4 (Fig 6C). It is possible that myostatin’s apparent tighter interaction with full length LTBP4 compared to the isolated amino-terminus of LTBP4 (compare myostatin content in lanes 7 and 9) may reflect a conformation only achievable with full length LTBP4. This is in agreement with previous data from Anderson et al. showing that the carboxy terminus of LTBP3 is not required for binding to myostatin [41]. We similarly assessed the binding site for GDF11 on LTBP4 and found an interaction with both the amino and carboxy terminal portions of LTBP4, although the interaction was more robust with the amino-terminal segment of LTBP4 (S7 Fig).

### Dystrophic mice overexpressing *LTBP4* have improved histopathology mediated through LTBP4 binding all three TGFβ isoforms

**Fig 7:**
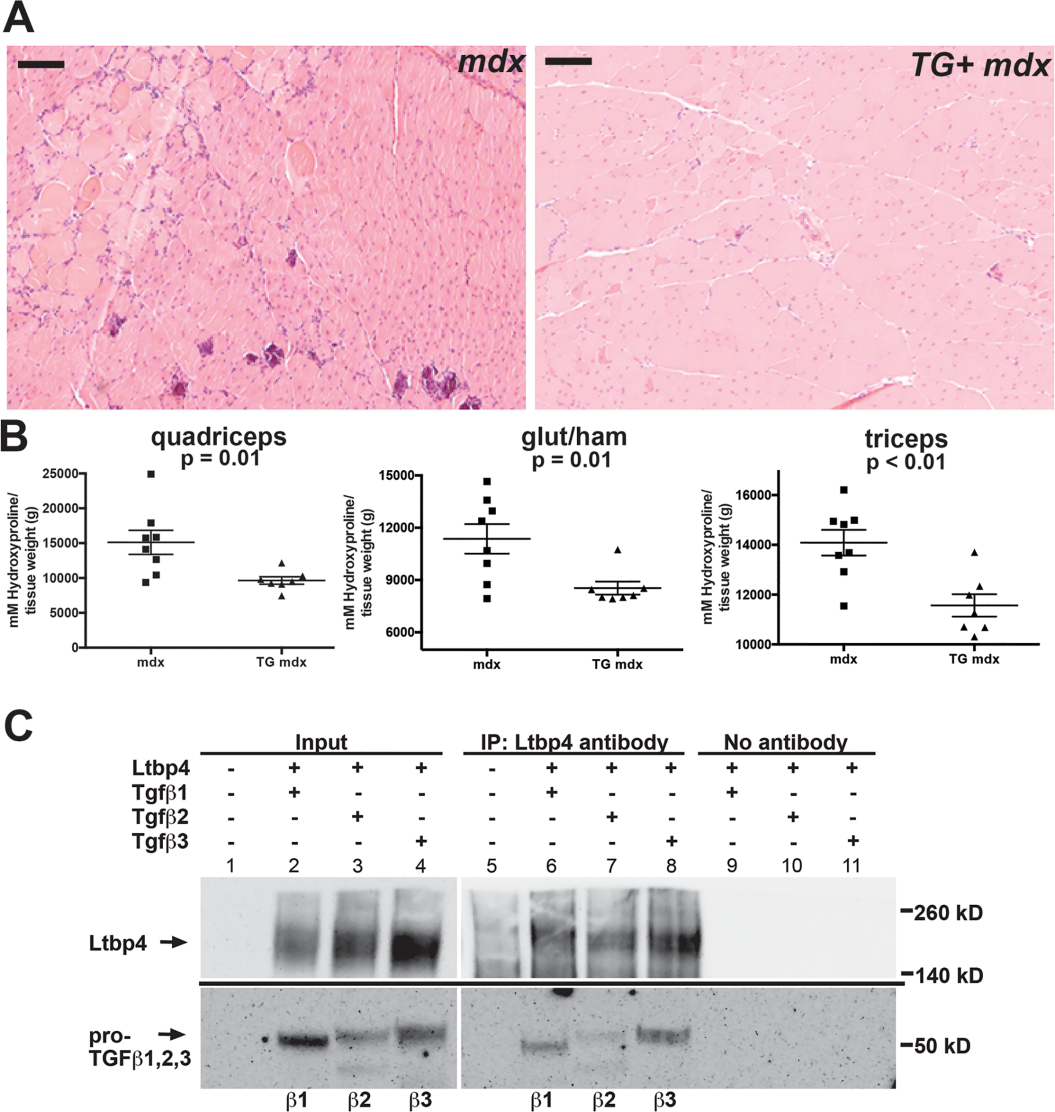
Improved histopathology and less fibrosis in LTBP4 TG+ *mdx* mice. **(A)** Quadriceps muscles from 8-week old animals were sectioned and stained with hematoxylin and eosin, showing improved histopathology of TG+ *mdx* mice compared to *mdx* controls. The region selected for imaging was selected from the image in S8 Fig (boxed). **(B)** The fibrosis content of TG+ *mdx* animals was assessed at 20 weeks using an assay for hydroxyproline, a modified amino acid found in collagen. Shown is hydroxyproline content from quadriceps, gluteus/hamstrings and triceps muscles, each showing reduction in hydroxyproline content in TG+ *mdx* (n = 7) compared to mdx (n = 8). **(C)** HEK293T cells were transfected to express heterologously mouse LTBP4 and either TGFβ1, TGFβ2, or TGFβ3. Co-immunoprecipitation was performed on cell lysates by immunoprecipitating with anti-LTBP4 antibody followed by immunoblotting with anti-myc antibody detecting TGFβ1, 2, and 3. A co-IP control for each experiment was performed without adding IP antibody. LTBP4 associated with all three TGFβ isoforms in vitro. Scale bar = 100μm.

LTBPs bind and sequester TGFβ in the extracellular matrix, regulating its availability to the TGFβ receptor (reviewed in [42, 43]). In muscular dystrophy, increased TGFβ signaling is thought to be detrimental, resulting in increased fibrosis (reviewed in [4]). Therefore, it was hypothesized that increased sequestration of TGFβ, mediated by higher levels of LTBP4 in TG+ mice, would result in a less severe form of muscular dystrophy. Hematoxylin and eosin stained muscle from TG+ *mdx* animals displayed improved histopathology compared to *mdx* controls (Fig 7A and S8 Fig). In addition to histology, we used quantitative measures of disease severity to assess transgenic mice at 20 weeks of age. Hydroxyproline content and Picro Sirius Red staining were used as measures of fibrosis, and Evans blue dye uptake was used to measure sarcolemmal leak. At 20 weeks, TG+ *mdx* mice had significantly less fibrosis than *mdx* mice as measured by hydroxyproline content (p = 0.001) (Fig 7B). Quadriceps muscles were fixed in formalin and stained with Picro Sirius Red to visualize collagen content. TG+ *mdx* muscles (n = 5) had significantly less Sirius Red staining compared to *mdx* mice (n = 4) when normalized to total fiber size (p = 0.0166, two-tailed student’s t-test) (S9 Fig). We also saw significantly less Picro Sirius Red staining in sections from quadriceps muscles from TG+ *mdx* mice (p<0.016) (Fig 7C). Evans blue dye uptake was not significantly different between TG+ *mdx* and *mdx* mice at 20 weeks, although TG+ *mdx* mice trended toward lower dye uptake (p = 0.2438), (S10 Fig). These results indicate that overexpression of *Ltbp4* ameliorates muscular dystrophy in mice through increased muscle mass and reduced fibrosis.

We tested all TGFβ1, TGFβ2, and TGFβ3 for their ability to interact with LTBP4. HEK293T cells were co-transfected with mouse LTBP4 and plasmids expressing mouse TGFβ1, TGFβ2 or TGFβ3. Immunoprecipitations were carried out using an antibody against LTBP4 followed by immunoblotting for the myc-epitope tag on TGFβ1, 2 or 3 (Fig 7D). TGFβ1, 2 and 3 each demonstrated a clear interaction with LTBP4 (lanes 6–8). RNA sequencing of mouse muscles showed that TGFβ1 was actually the lowest expressing TGFβ isoform in skeletal muscle, with TGFβ2 expressing approximately twice the transcript level of TGFβ1, and TGFβ3 expressing the highest, approximately 3–6 times more than TGFβ1 (Table 1). The varying levels and expression patterns of TGFβ1, 2 and 3, which we here show interact with LTBP4, are likely critical during injury and repair.

## Discussion

### Overexpression of LTBP4 in muscle improves muscular dystrophy

LTBP4 is an extracellular matrix protein that was previously identified using an unbiased genomewide scan for modifiers of muscular dystrophy [22]. The genetic mapping studies in mice took advantage of the DBA2/J strain of mice, which was ultimately shown to harbor a deletion that altered a hinge region of the LTBP4 protein [44]. Within muscle, especially diseased muscle, LTBP4 protein is likely produced by multiple different cell types, including myofibers and fibroblasts. We now expressed LTBP4 protein in mature myofibers using a muscle specific promoter and found that overexpression of LTBP4 protein mitigates aspects of muscular dystrophy in the *mdx* mouse model. The expression of LTBP4 protein tightly apposed to the sarcolemma in a costameric pattern positions LTBP4 to mediate signaling directly within muscle. *Ltbp4* has an essential role in development [20], but LTBP4 protein overexpression was not toxic to muscle leading to an expansion of largest myofibers in the wildtype background. The results were most evident in muscles of the forelimb and of a smaller magnitude than what is seen in *Mstn* null mice. This *Ltbp4* transgene was driven by the human skeletal actin promoter and was not observed to express at appreciable levels in diaphragm muscle, although others have observed expression from this same promoter in diaphragm muscle, for example when assessing mini-dystrophin expression [45]. Variable levels of *Ltbp4* transgene expression may account for differential effects in across muscle groups.

Interestingly, the effect of LTBP4 protein overexpression was enhanced in the setting of muscular dystrophy compared to what was seen in the wildtype background. Overexpression of LTBP4 protein in the *mdx* mouse model produced an overall effect on the animal’s size, not only increasing muscle mass but also increasing kidney and heart size relative to *mdx* mice without the transgene. Organ growth in this setting was proportional to animal size and did represent selective organ hypertrophy. Organ growth was likely an indirect result since *Ltbp4* expression in this context was muscle-specific. It is possible that muscle-derived cytokines contribute to other organ growth. Increased cardiac mass can be either pathological or physiological. In this setting, where LTBP4 protein is being expressed in skeletal muscle, the increase in other organs is likely an indirect consequence that could be mediated by circulating myostatin or other, as yet unknown, molecules. It was previously noted that conditional deletion of *Mstn* in the heart ablated skeletal muscle wasting in the context of heart failure, suggesting that organ specific production of myostatin can have remote effects [46].

### LTBP4 is multi-scaffolding matrix protein

LTBPs are known to interact with the primary TGFβ family members TGFβ 1–3 and are also thought to act as chaperones for ensuring efficient processing and extracellular deposition of these proteins [33]. We now found that LTBP4 interacts with TGFβ1, TGFβ2 and TGFβ3. These results are in contrast to a previous study that found LTBP4 was only able to bind TGFβ1, however this group did not use full length LTBP4 protein, which may be necessary for efficient binding [40]. We attribute the increased muscle fiber area seen in *Ltbp4* TG+ *mdx* mice to the regulation of myostatin by LTBP4. The reduction in fibrosis may be due to sequestration of myostatin as well as TGFβ, since reduction in active myostatin or TGFβ would exert this same effect of reducing fibrosis [4, 47]. Notably, we found that myostatin and TGFβ bind distinct subdomains of LTBP4 and it is possible that LTBP4 may bind both of these proteins simultaneously. Whether such binding could be competitive or cooperative is unknown but the effect of increased mass combined with decreased fibrosis suggests that LTBP4 acts through both these mechanisms in the muscular dystrophy disease state.

*Mdx* mice deficient in myostatin have an improved muscular dystrophy phenotype and increased mass compared to *mdx* mice [32]. Li et al. found that treatment of muscle fibroblasts with myostatin resulted in increased proliferation and that injection of myostatin directly into mouse muscle induces fibrosis [47], consistent with a direct effect of myostatin on fibroblasts. The degree to which of the TGFβ isoforms or myostatin contributes to the reduction of fibrosis is not clear since even muscle derived expression of this matrix associated protein may act locally on fibroblasts. Reducing TGFβ activity through antibody neutralization has beneficial effects on the *mdx* mouse phenotype [48]. Ligand traps have been developed to sequester or neutralize myostatin and are actively being pursued in clinical trials [49]. In mouse models, where myostatin inhibition has been well studied, the increase in muscle mass was associated with an absolute increase in strength or force, rather than an increase in specific force [50]. Thus, the increase in muscle size is associated with a proportional increase in strength, as was seen here. Other approaches to reduce myostatin signaling include administration of the myostatin inhibitory prodomain as well as soluble receptors (reviewed in [51–54]). Approaches to upregulate LTBP4 may be useful since they would target both myostatin and TGFβ.

We found that LTBP4 also binds GDF11. GDF11 has received attention for its role in the aging heart and muscle [9, 38]. The current study investigates the role of LTBP4 overexpression in a model of muscular dystrophy. Because LTBP4 binds GDF11 in vitro, it is possible that muscle specific LTBP4 overexpression could alter sarcopenia or other aging related phenotypes. The current studies were not carried out of sufficient duration to draw any conclusions on aging-related phenotypes in mice. Additionally it may be necessary to express LTBP4 outside of skeletal muscle to alter aging. It has been suggested that GDF11 levels decline with age and that supplementation may be beneficial. However, other groups have not found a decline of GDF11 with age [10, 39]. The similarity between myostatin and GDF11 is striking, especially in their active domains where there is near complete identity. The capacity to distinguish these moieties based on binding partners, including antibodies or other protein-protein interactions is challenging given the similarity between GDF11 and myostatin. Therefore the significance of LTBP4 binding to GDF11 is not entirely clear. We used RNA sequencing to estimate the relative abundance of GDF11 and myostatin, and this method may not reflect protein levels. Nonetheless, a near 10:1 ratio of myostatin to GDF11 mRNA levels suggests that myostatin may predominant in muscle and that LTBP4 exerts its effect more through myostatin rather than GDF11.

LTBP4 role as multi-cytokine regulator may extend to other members of the TGFβ superfamily of proteins, including BMPs. Studies of acute injury in flies, mice, and humans show increased expression levels of multiple cytokines and it is possible that this effect could be mediated by LTBPs [36, 55, 56]. Furthermore, targeting LTBP4 as a therapeutic may have effects on multiple TGFβ superfamily members.

## Materials and Methods

### Ethics statement

Mice were housed in a specific pathogen free facility in accordance with Institutional Animal Care and Use Committee (IACUC) regulations. Euthanasia was performed through carbon dioxide or anesthetic gas inhalation followed by cervical dislocation and removal of the heart. All methods using living animals in this study were performed in ethical accordance with the American Veterinary Medical Association (AVMA) and under protocols fully approved by both the Institutional Animal Care and Use Committee (IACUC) at the University of Chicago (protocol 70619) and the IACUC at Northwestern University Feinberg School of Medicine (protocol number ISO00000911). Consistent with the approvals stipulated by these protocols, all efforts were made to minimize suffering.

### Generation of transgenic mice

Mouse *Ltbp4* (uc009fvt.2, transcript variant 1) was ligated downstream of the human skeletal actin (HSA) promoter and human troponin enhancer for high-level expression specifically in mature myofibers [26, 27]. The HSA-*Ltbp4* construct was injected into fertilized oocytes from C57BL6NCrl mice by the University of Cincinnati transgenic core facility. Transgenic mice were bred with C57BL6 littermates or *mdx-/-* females (C57BL/10ScSn-Dmdmdx/J, JAX #001801). Control mice were generated by breeding C57BL/6J (JAX # 000664) littermates or by crossing C57BL/6J males x *mdx*-/- females (JAX #001801).

### Genotyping

Transgenic mice were genotyped by PCR of tail DNA, using primers spanning exons 5–11 of *Ltbp4*: Forward: 5’ GTTTATACAATGCCACTAGCCAACCA 3’; Reverse: 5’ TGTATCGGAGGTCAGAAGCTGAATAG 3’. Genotyping for *mdx* mice was done by PCR with primers spanning exon 23 of dystrophin: Forward: 5’ GAAACTCATCAAATATGCGTGTTAGTG 3’; Reverse: 5’ AGTGCCCCTCAATCTCTTCAAATTCTG 3’. The *mdx* PCR products were purified with ExoSAP-IT reagent (USB) and sequenced by Sanger sequencing at the University of Chicago sequencing core.

### Grip strength

Grip strength tests were done 48 hours prior to sacrifice, prior to Evans Blue Dye injection. Grip strength was performed using a Chantillon Ametek force transducer in a Columbus Instruments (Columbus, OH) apparatus. Mice were held by the tail, allowed to grasp a triangular bar with both forepaws, and pulled away from the bar horizontally until they lost their grasp. Force was recorded for 10 trials; values reported are the average of these trials. The same operator, who was blinded to genotype, conducted all measurements. Results are reported as kgf, kilogram force.

### Muscle collection and preparation

Mice were euthanized at various ages in accordance with IACUC. Skeletal muscles (quadriceps, triceps, gluteus/hamstrings, gastrocnemius/soleus, abdominal, diaphragm muscles) and the heart (right ventricle, left ventricle) were dissected, weighed and subsequently used for study. Whole tissue lysis buffer (50mM HEPES pH 7.5, 150mM NaCl, 2mM EDTA, 10mM NaF, 10mM Na-pyrophosphate, 10% glycerol, 1% triton X-100, 1X Roche cOmplete protease inhibitor tablet) and a dounce tissue grinder were used to homogenize tissue samples for immunoblotting.

### Microsomal preparation

Fractionation of cell lysates was performed following the protocol of (Ohlendieck and Campbell, 1991) with modifications as described [57]. All fractions were stored at -80°C. For co-IP experiments, fresh lysates were used without freeze-thaw cycles.

### Evans Blue dye uptake assay

Evans Blue dye (EBD) uptake was measured as described previously [58] with modifications as described [59]. Multiple muscle groups were assessed including the abdominal, diaphragm, quadriceps, gastrocnemius/soleus, gluteus/hamstrings, and triceps and normalized to tissue weight. Absorbance was measured at 620 nm on a spectrophotometer. Results are reported as arbitrary OD units/g of tissue.

### Hydroxyproline assay

The content of hydroxyproline in each tissue was assayed as described previously [22, 60]. Skeletal muscle tissues (quadriceps, abdominal (rectus femoris), gastrocnemius/soleus, gluteus/hamstrings, and triceps) were assayed. A standard curve was created using 0–5000 nMol hydroxyproline (Sigma H-5534) as starting material and the zero mM standard was used as a blank when measuring absorbance. Amount of hydroxyproline in each tissue was normalized to tissue mass, and the results are reported as nMol hydroxyproline/g tissue.

### Histological analyses

Quadriceps muscles were fixed in formalin for 48 hours, embedded in paraffin, and sectioned at 6μm thickness. Sections were stained with hematoxylin and eosin and Picro Sirius Red. Slides were imaged using a Zeiss Axiocam epifluorescence microscope and iVision or ZEN Pro software. Picro Sirius Red sections were quantified for the signal density emanating from collagen and normalized to the total fiber size. Fiber size measurements were taken from 4 immediately adjacent fields taken at 10x, fibers were outlined in Adobe Photoshop and fiber sizes were determined using ImageJ. Statistical analyses were done using GraphPad Prism. Analyses were done blinded to genotype.

### Confocal immunofluorescence microscopy

Gluteus and hamstring muscles were dissected as a unit and placed into relaxing solution (100mM BES, 15mM creatine phosphate, 5mM dithiothreitol, 17mM propionic acid, 4.74mM adenosine triphosphate, 7mM ethylene glycol tetraacetic acid, 5.43 mM magnesium chloride, 0.02mM calcium chloride, adjusted to pH 7.2 with sodium hydroxide), modified from [61]. While in relaxing solution, small fiber bundles of 2–4 fibers were teased, isolated, and mounted onto slides. Fibers were rinsed once in phosphate buffered saline and incubated in primary antibody overnight at 4°C. Fibers were rinsed 3x in PBS and incubated in secondary antibody overnight at 4°C. Fibers were rinsed 3x in PBS, and after aspiration of PBS, DAPI was applied and coverslips were placed over the fibers. Mounting media with DAPI (Vector Labs) was used to mount sections. A control was performed using no primary antibody. Slides were imaged using a Leica TCS SP2 AOBS laser scanning confocal microscope.

The chicken polyclonal anti-LTBP4 was previously described antibody as raised to a peptide (EPRPEPRPDPRPGPELP) and was used at 1:250 [30]. The secondary antibody to detect LTBP4 was Alexa Fluor 488 donkey anti-chicken IgY (Jackson Immunoresearch). Texas Red-X phalloidin (Life Technologies) was used at 1:500. WGA-Alexa 488 (Life Technologies, W11261) was used at 10 μg/ml. Anti-laminin polyclonal antibody (Sigma, L9393) was used at 1:50 dilution. Donkey anti-rabbit IgG coupled to Alexa 594 (Invitrogen, A-21207) was used as a secondary antibody at 1:500 to detect laminin. Antibodies were diluted in blocking buffer (5% fetal bovine serum in PBS) for use.

### Expression constructs

The long isoform of mouse *LTBP4-L* (uc009fvt.2, transcript variant 1) was ligated into the expression vector pcDNA3.1/V5-His-TOPO (Invitrogen) and engineered to include an Xpress epitope tag in frame at the immediate carboxy terminus. Mouse cDNA clones of *Mstn* (NM_010834), *Gdf11* (NM_010272), *Tgfβ1* (NM_011577), *Tgfβ2* (NM_009367), *and Tgfβ3* (NM_009368) were purchased from Origene (catalog numbers MR227629, MR223819, MR227339, MR225633, and MR206441, respectively). A myc-DDK tag was included in frame at the extreme carboxy teriminus (3’) end of each open reading frame. Full length human LTBP4 (NM_001042544.1), MSTN (NM_005259.1), and myc-tagged GDF11 (NM_005811) cDNA clones were purchased from Origene (catalog numbers SC311430, SC124056, and RC222080, respectively). The Xpress epitope (DLYDDDDK) was added onto the 5’/N-terminus of human LTBP4. Primers encoding the Myc epitope tag (EQKLISEEDL) were used to add this sequence to the 3’/C-term of MSTN. Amino and carboxy terminal clones corresponding to exons 1–12 and exons 11–34 of human *LTBP4* were cloned and engineered to contain Xpress tags on the amino termini. Schematics of the constructs are shown in S11 Fig.

### Cell culture

HEK293-T cells were obtained from ATCC (catalogue number CRL-11268). Cells were grown in Dulbecco's Modified Eagle Medium (DMEM) supplemented with 10% fetal bovine serum (Invitrogen, lot #1420768) and 1% penicillin/streptomycin (Invitrogen) in 5% CO_2_.

### Transfections

HEK293-T cells were plated at 1.5x10^6^ cells per well in 6-well plates the day prior to transfection. Media was changed to Opti-MEM Reduced Serum Media prior to transfection. Transfections were performed using FuGENE HD transfection reagent (Promega) at a DNA:FuGENE ratio of 1:3. Opti-MEM was replaced with serum-rich media 24 hours post-transfection and cells were harvested 3 days post-transfection.

### Co-immunoprecipitation

Cultured 293T cells were rinsed once with ice-cold phosphate buffered saline and lysed with 150μl immunoprecipitation lysis buffer (150 mM NaCl, 50 mM Tris HCl (pH 8), 25 mM β-glycerophosphate, 10 mM sodium pyrophosphate, 1x cOmplete Protease Inhibitor Tablet (Roche), 1 mM phenyl-methylsulfonyl fluoride, 2 mM EDTA, and 0.1% Triton X-100), (modified from Anderson et al. 2008) [41]. Cells were scraped from the plate using a chilled cell-lifter and transferred to Eppendorf tubes. Lysates were mixed by pipetting up and down, followed by centrifugation at 14,000 *g* for 5 min at 4°C to remove cellular debris. The protein concentration of the supernatant was determined using the Bio-Rad protein assay. Five hundred μg protein lysate was precleared with 45μl Protein G Plus/ Protein A-agarose suspension (Calbiochem) for one hour. Pre-cleared lysates were incubated with 3μg anti-LTBP4 antibody for 3 hours, followed by incubation with 45μl Protein G/A agarose bead slurry for 2 hours. Beads were washed with lysis buffer and centrifuged at 3,000 *g* (3 x 3 minute washes). Following the final wash, beads were eluted using 2X Laemmli sample buffer containing β-mercaptoethanol and boiled for 5 minutes. Prior to boiling, all steps were done at 4°C. Immunoprecipitation from muscle was carried out on lysates of combined triceps, quadriceps, gluteus, hamstring, and gastrocnemius muscle groups. Muscles were homogenized in a tissue lysis buffer (20mM sodium pyrophosphate, 20mM sodium phosphate monohydrate, 1mM MgCl2, 0.303M sucrose, 0.5mM EDTA, 1mM PMSF, 1X Roche COMPLETE protease inhibitor tablet) using a Tissue Tearor Homogenizer. Homogenized tissues were further lysed with Dounce tissues grinders and 50μl lysate was saved for an input control. One mg of muscle lysate was brought to a total volume of 500μl with immunoprecipitation lysis buffer, pre-cleared with 45 μg Protein G Plus/ Protein A-agarose, and from this point treated in the same manner as cell lysates for co-immunoprecipitation. Three μg anti-myostatin antibody (EMD Millipore, #AB3239-I) was used for immunoprecipitation.

### Immunoblotting

The protein concentration of the cell or muscle lysate was determined using the Bio-Rad Protein assay (Bio-Rad). Proteins were separated on either a 6% SDS-polyacrylamide gel (anti-Xpress blot) or a 4–20% polyacrylamide pre-cast gel (Pierce #25204) (all other blots) and transferred to PVDF Immobilon-P membrane (Millipore). Blocking and antibody incubations were done using StartingBlock T20 (TBS) blocking buffer (Pierce). Rabbit polyclonal anti-LTBP4 antibodies were generated as described [30]; LTBP4 and anti-Xpress antibodies were raised against the epitopes EPRPEPRPDPRPGPELPLP and DLYDDDDK, respectively, and purified by Pocono Rabbit Farm and Laboratory. Primary antibodies used are as follows: mouse monoclonal anti-Myc (Millipore #05–724) at 1:4000, mouse monoclonal anti-myostatin (Pierce, #MA5-15486) at 1:1000, rabbit polyclonal anti-myostatin (EMD Millipore, #AB3239-I) at 1:1000, and rabbit polyclonal anti-Xpress at 1:500, rabbit polyclonal anti-LTBP4 at 1:500, and mouse monoclonal anti γ–tubulin (Sigma #T6557) at 1:10,000. Goat anti-mouse and goat anti-rabbit secondary antibodies conjugated to horseradish peroxidase (Jackson Immunoresearch #115-035-003 and #111-035-003) were used at a dilution of 1:8000. Clarity ECL substrate (Bio-Rad) was applied to membranes and membranes were visualized using a Biospectrum Imager (UVP). MemCode membrane stain (Pierce) was used to stain the entire blot to ensure complete transfer, and the 43 KDa band from this protein staining representing actin was used as a loading control. Immunoblot bands were quantified using ImageJ gel analysis tools followed by statistical analysis in GraphPad Prism.

### Phylogenetic tree construction

Multiple Sequence Alignment was done for mouse TGFβ family members using Clustal Omega (ClustalO). A phylogenetic tree was derived from these protein alignments using the Interactive Tree of Life (iToL) platform [62].

### RNA sequencing

RNA sequencing libraries were constructed using the Illumina TruSeq RNA Sample Prep Kit version 2.0 on abdominal muscles from 129T2/SvEmsJ and DBA/2J strains of mice. Three RNA samples from each strain were indexed with unique adapters and pooled for 100bp paired-end sequencing with Illumina HiSeq 2000. RNA-seq reads were aligned with TopHat v2.0.2 to mouse genome assembly mm9 [63]. Transcripts were assessed and quantities were determined using FPKM values by Cufflinks and Cuffdiff package [64].

## Supporting Information

S1 Fig The HSA transgene does not express in diaphragm muscle. (PDF)

S2 Fig WGA staining of WT and LTBP4 TG muscle. (PDF)

S3 Fig Fiber area and distribution in WT and LTBP TG muscle. (PDF)

S4 Fig Enlargement of LTBP4 TG muscle. (PDF)

S5 Fig Comparison of muscle and organ mass in mdx and TG+ mdx animals. (PDF)

S6 Fig Quantitation of myostatin in mdx and TG+ mdx muscle. (PDF)

S7 Fig GDF11 binding to LTBP4. (PDF)

S8 Fig Improvement in histopathology TG+ mdx muscle. (PDF)

S9 Fig Improvement in fibrosis in TG+ mdx muscle by Sirius red. (PDF)

S10 Fig Evans blue dye uptake in TG+ mdx compared to mdx muscle. (PDF)

S11 Fig Summary and schematic of expression constructs. (PDF)
