# Supplementary figures and images for: Overexpression of Latent TGFβ Binding Protein 4 in Muscle Ameliorates Muscular Dystrophy through Myostatin and TGFβ
Source: PLoS Genet. 2016 May 5;12(5):e1006019. doi: 10.1371/journal.pgen.1006019 (PMC4858180; doi:10.1371/journal.pgen.1006019)

**S1 Fig. The HSA promoter expresses in limb skeletal muscles but not diaphragm muscle.**

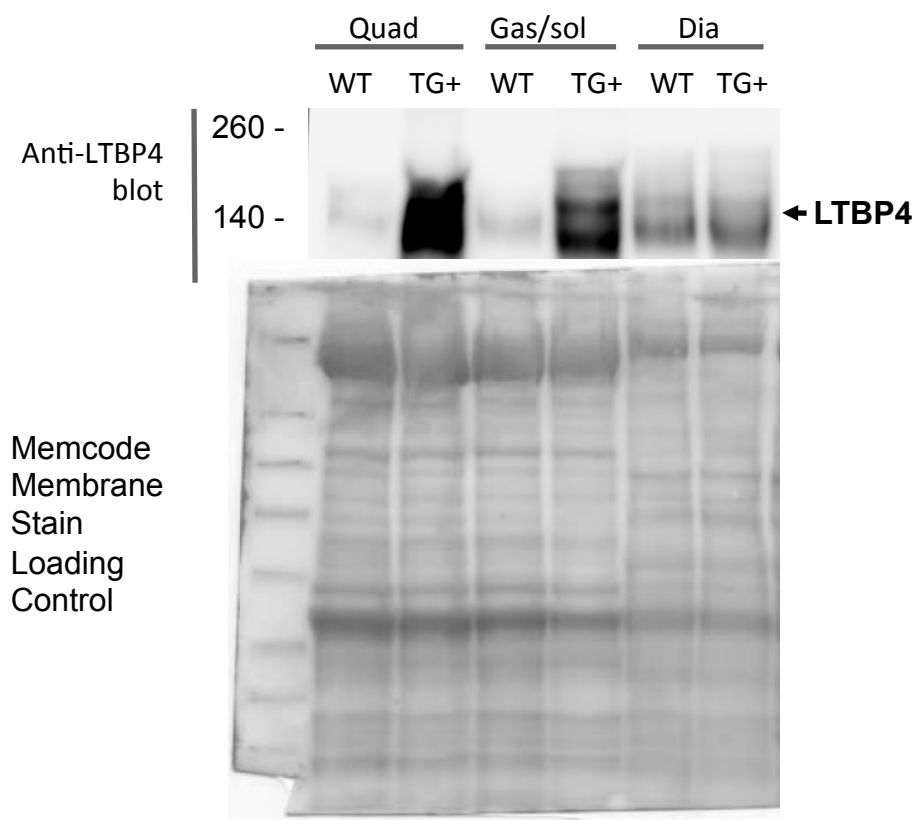

Supplement: S1 Fig — (PDF) [file pgen.1006019.s001.pdf]

S2 Fig. WGA binding to WT and Tg + muscle

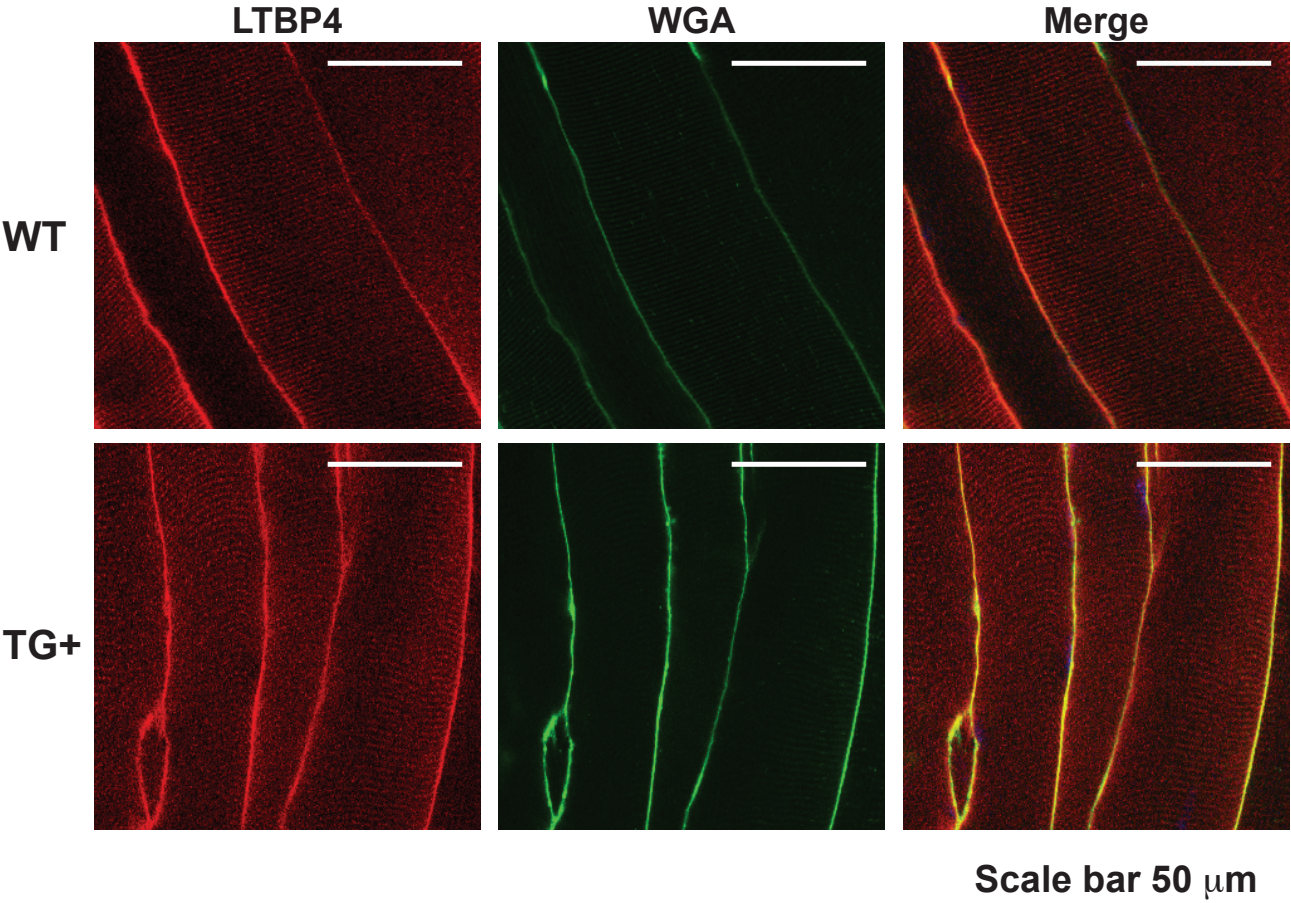

Supplement: S2 Fig — (PDF) [file pgen.1006019.s002.pdf]

**S4 Fig. Eight week muscle mass in WT vs LTBP4 TG+.**

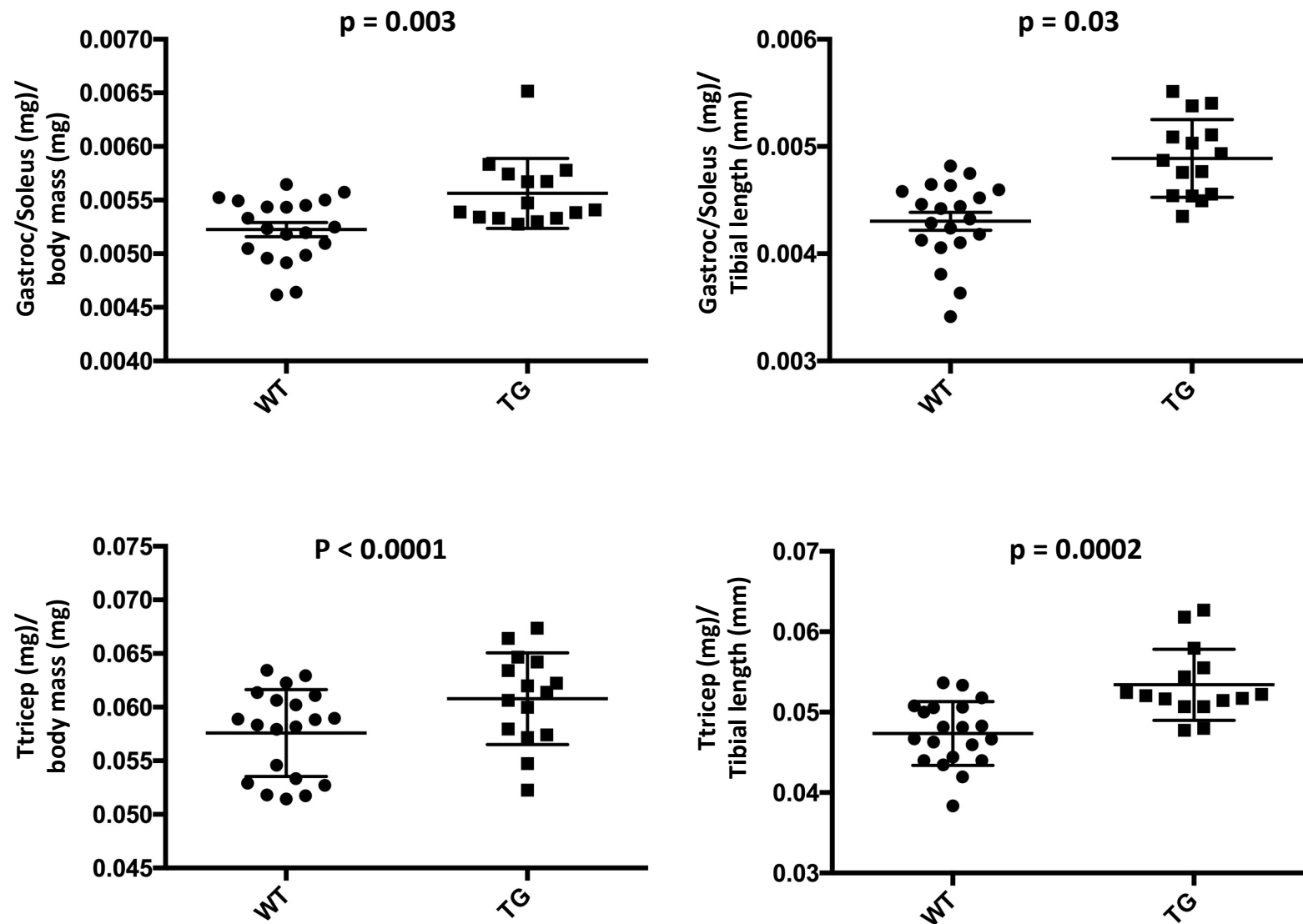

Two tailed, unpaired t test, Bar = SEM

Supplement: S4 Fig — (PDF) [file pgen.1006019.s004.pdf]

S5 Fig. Mass comparison of mdx vs TG+ mdx mice (34 weeks)

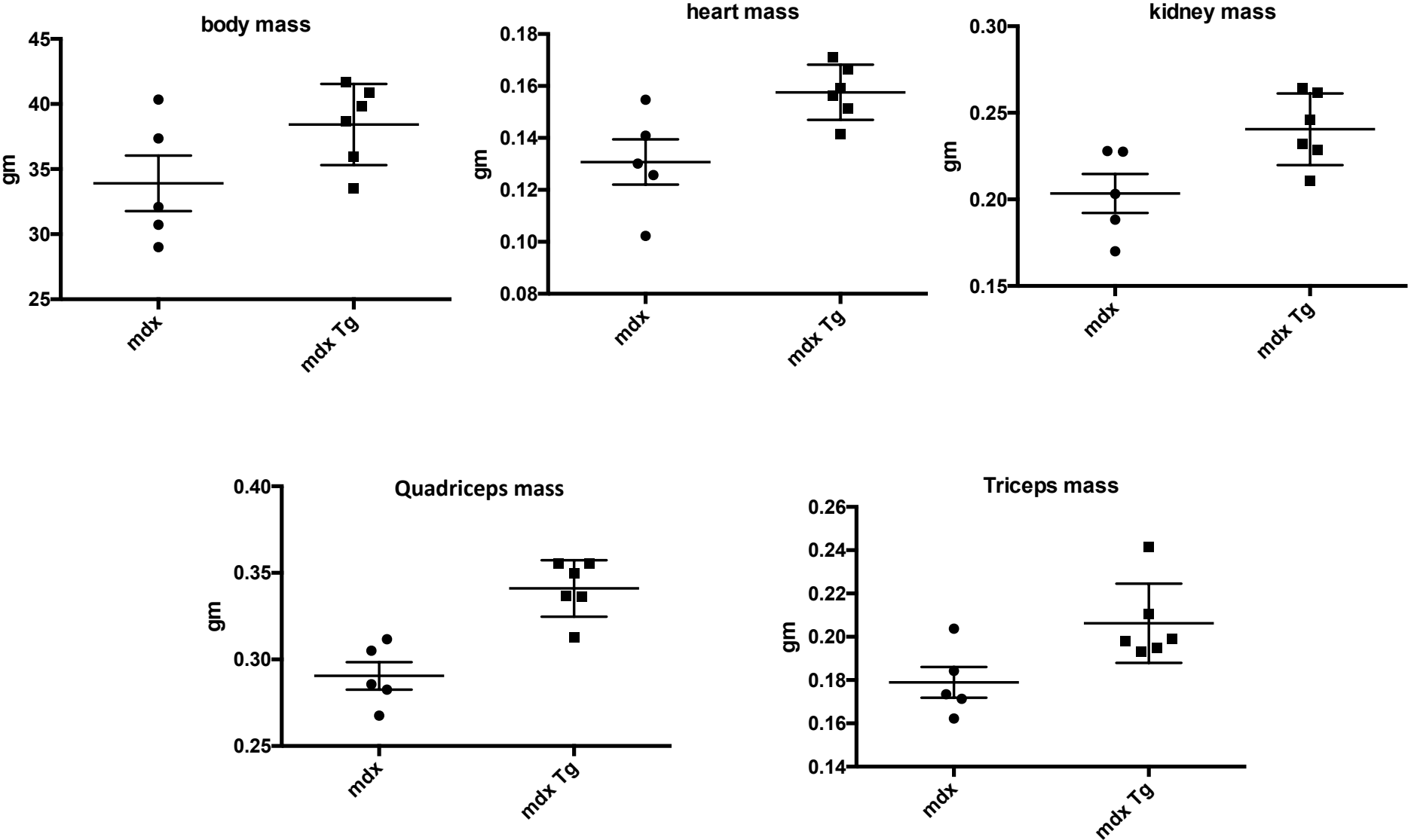

Bar = SEM

Supplement: S5 Fig — (PDF) [file pgen.1006019.s005.pdf]

S6 Fig. Myostatin in muscle fractionation.

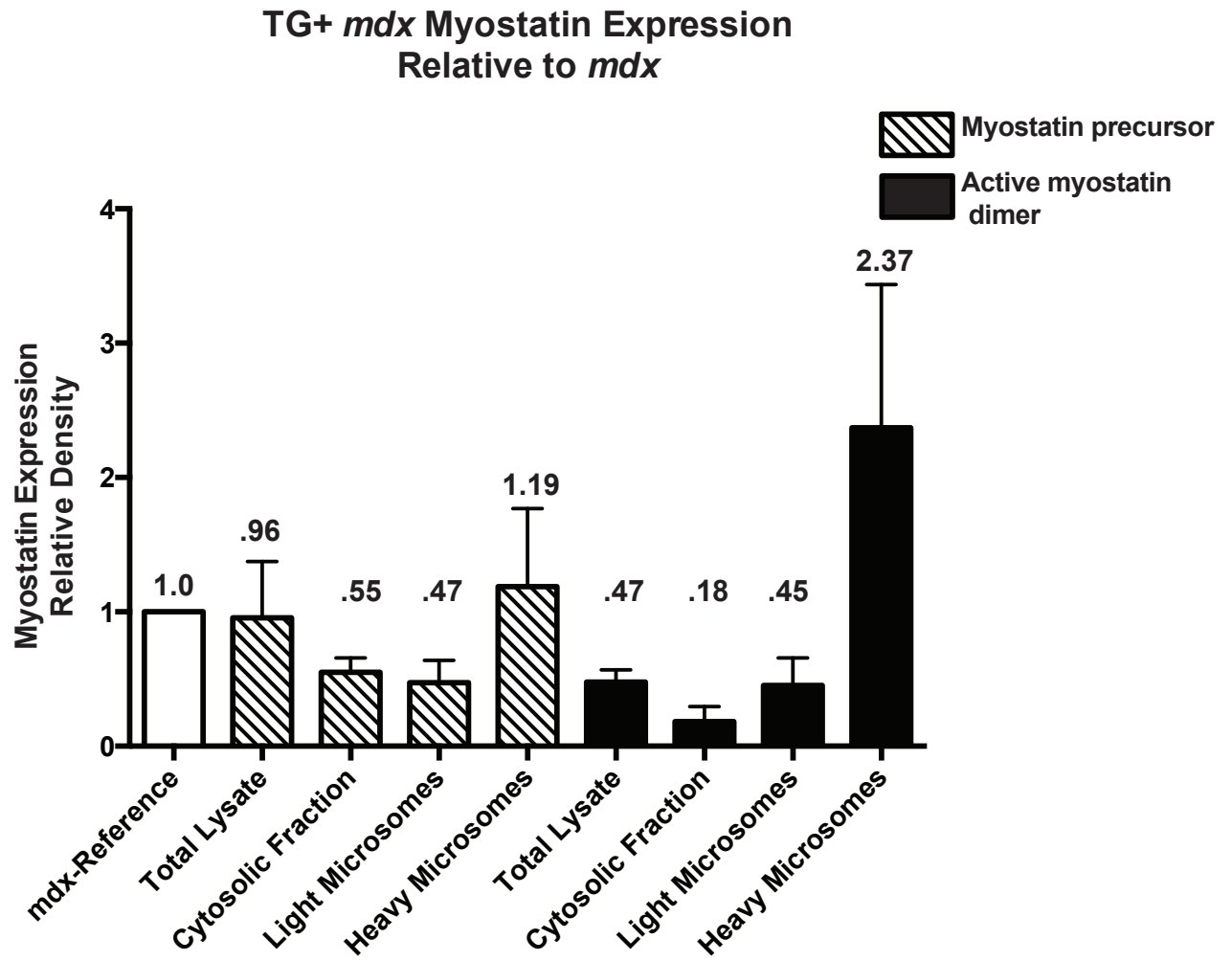

Supplement: S6 Fig — (PDF) [file pgen.1006019.s006.pdf]

## S8 Fig. Quadriceps muscle histology.

**mdx**

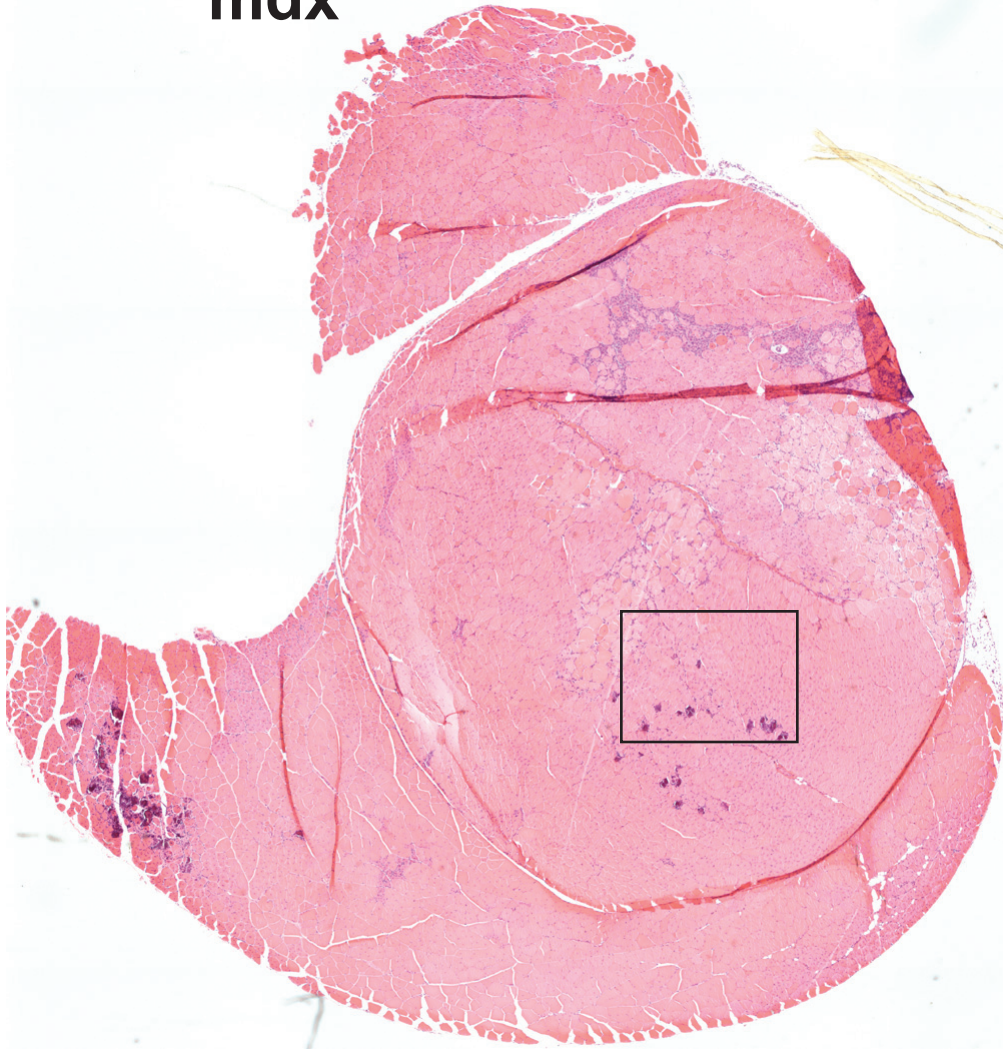

**mdx Tg**

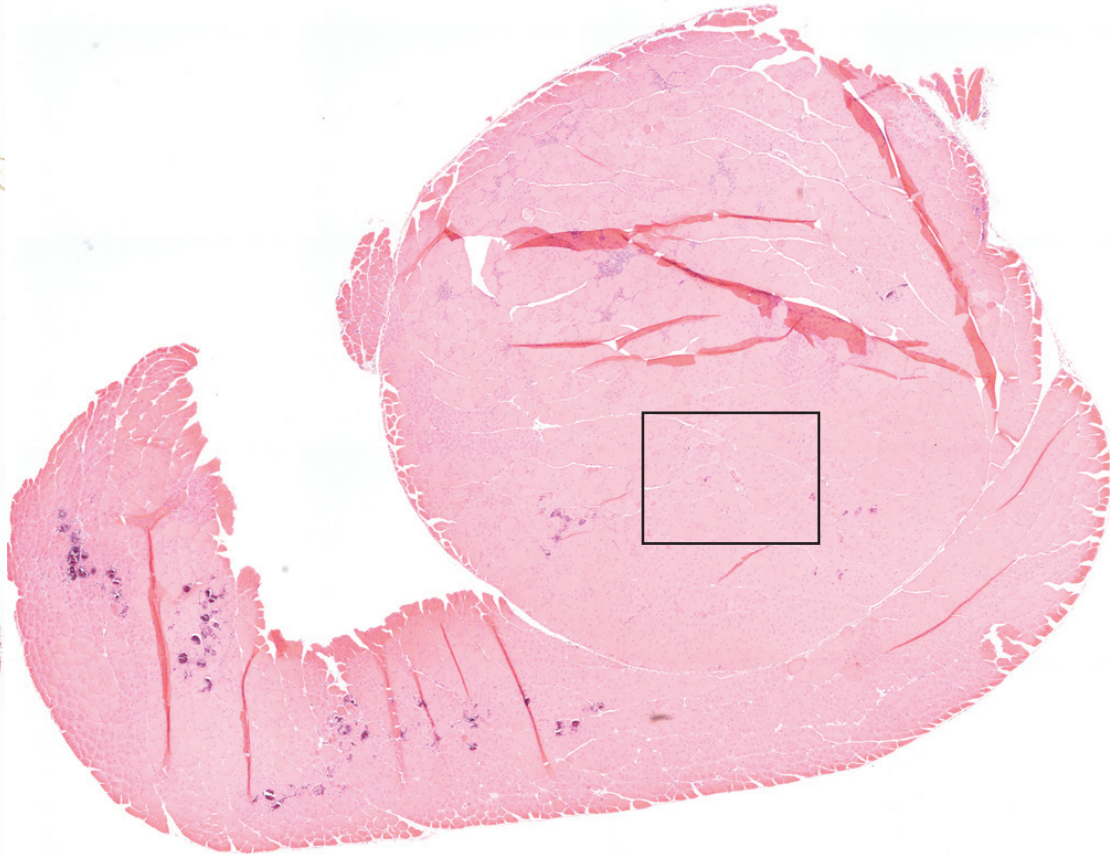

Supplement: S8 Fig — (PDF) [file pgen.1006019.s008.pdf]

**S9 Fig. Sirius Red**  
**mdx,Tg**

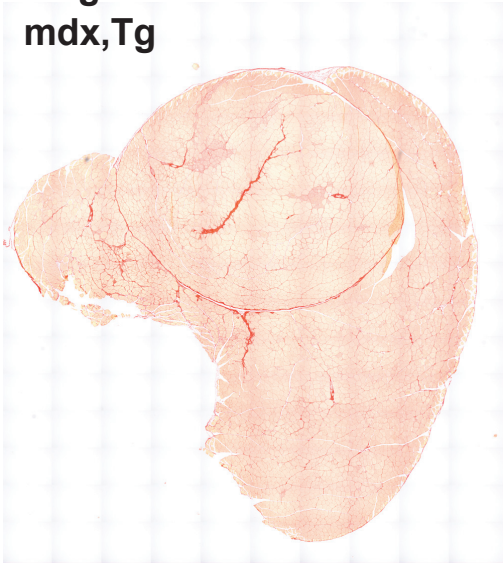

**mdx,Tg**

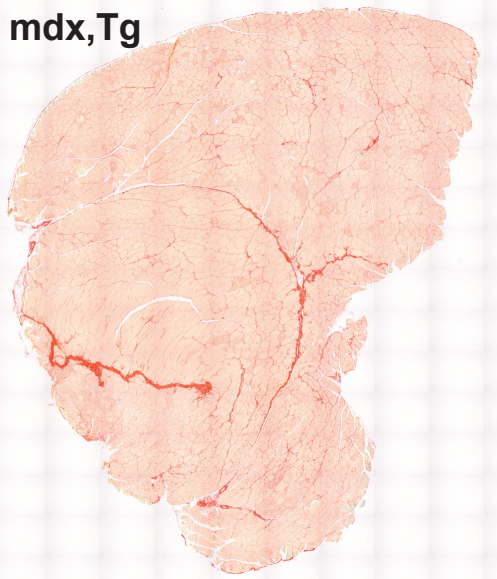

**mdx,Tg**

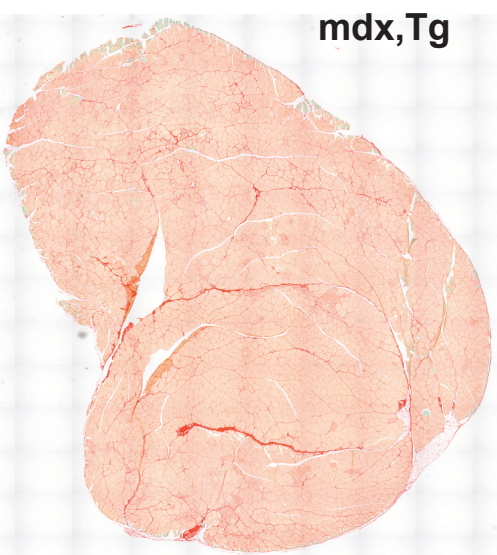

**mdx,Tg**

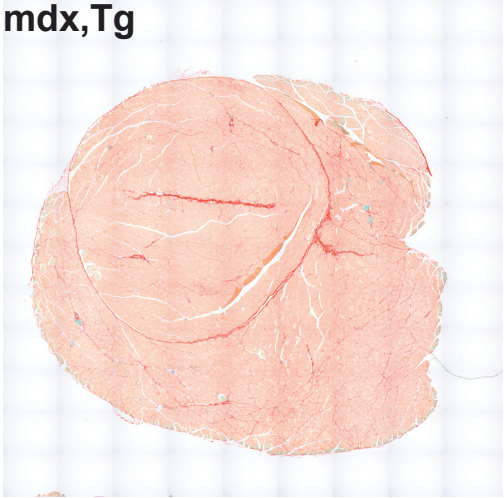

**mdx,Tg**

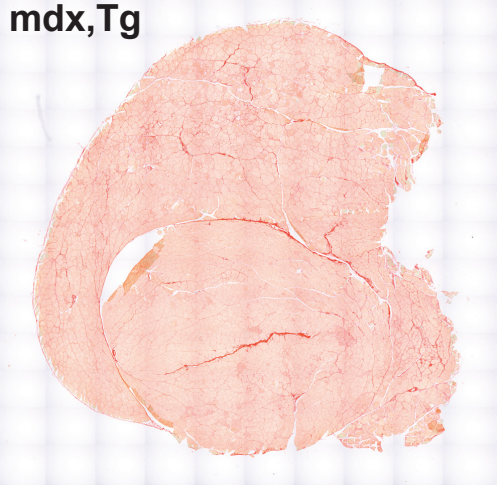

**mdx**

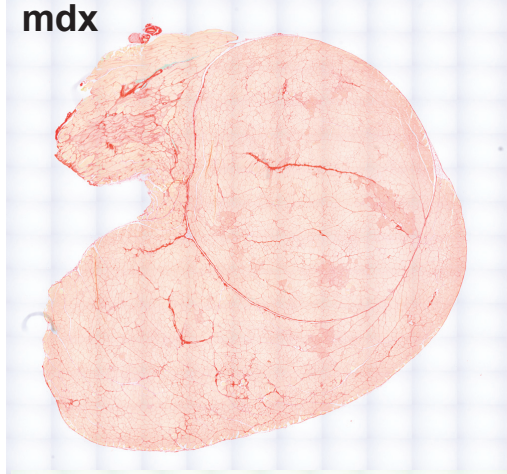

**mdx**

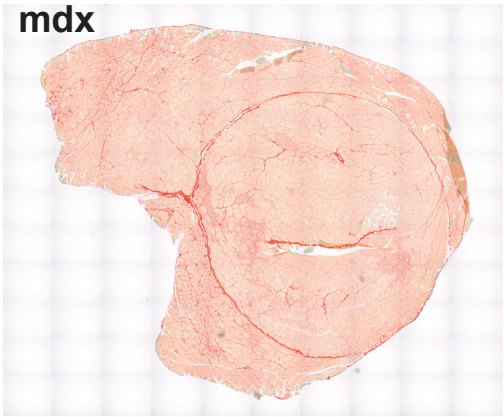

**mdx**

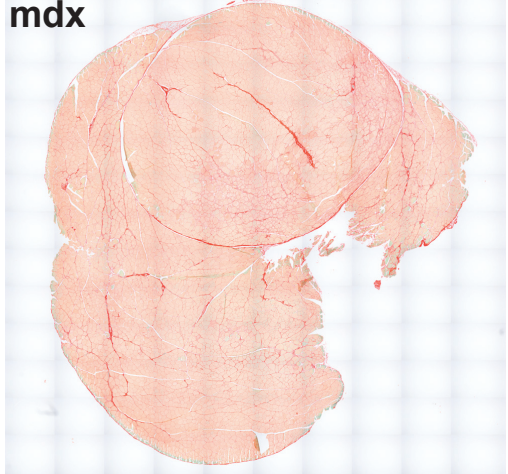

**mdx**

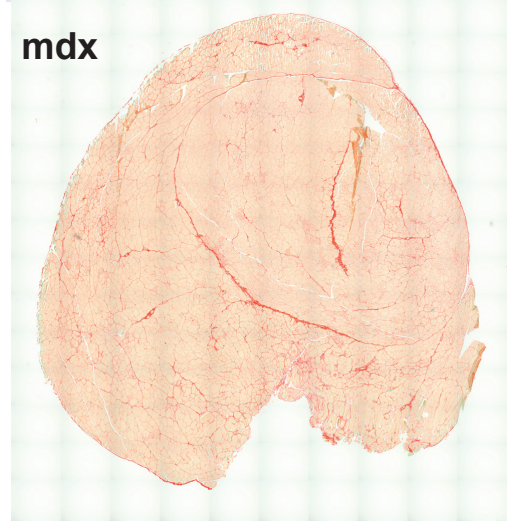

**Sirius Red Staining**

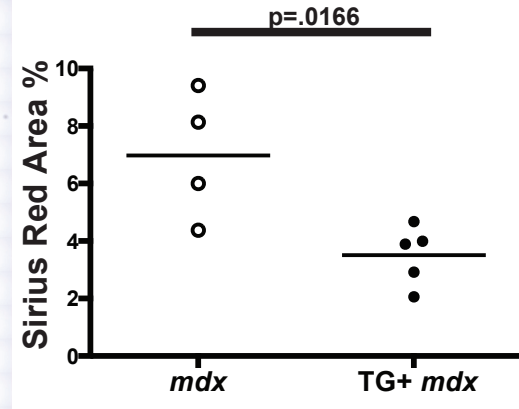

Supplement: S9 Fig — (PDF) [file pgen.1006019.s009.pdf]

**S10 Fig. Evans Blue dye is not significantly changed between mdx and TG+ mdx.**

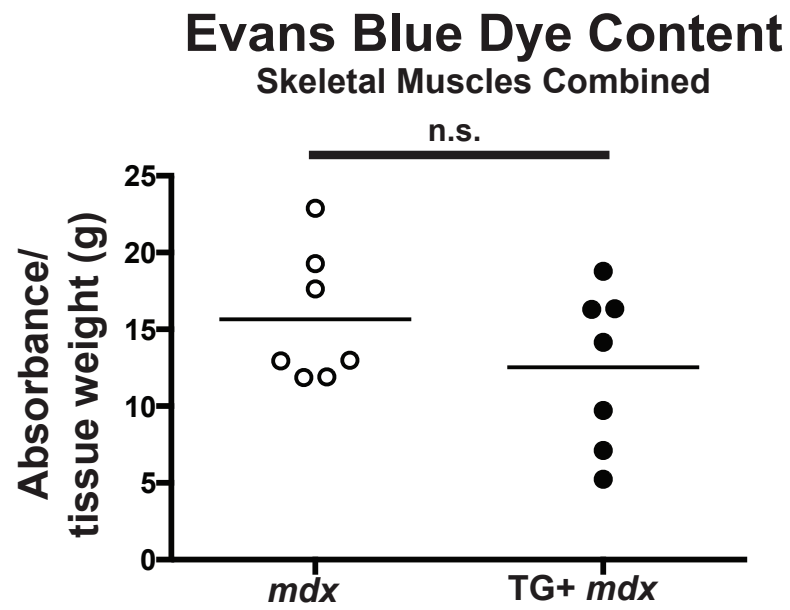

Supplement: S10 Fig — (PDF) [file pgen.1006019.s010.pdf]
