# Supplementary material for: Overexpression of Latent TGFβ Binding Protein 4 in Muscle Ameliorates Muscular Dystrophy through Myostatin and TGFβ
Source: PLoS Genet. 2016 May 5;12(5):e1006019. doi: 10.1371/journal.pgen.1006019 (PMC4858180; doi:10.1371/journal.pgen.1006019)

**S3 Fig. Fiber area is unchanged in WT vs LTBP4 TG+ quadriceps muscle. TG+ quadriceps has an increase in the largest fibers, those >6500 μm2.**

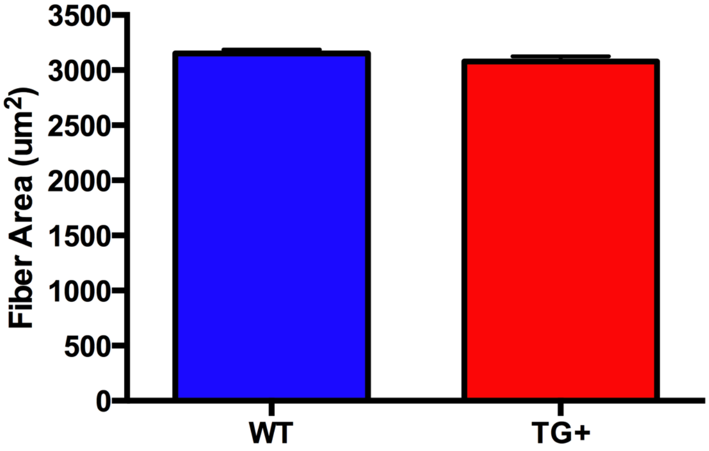


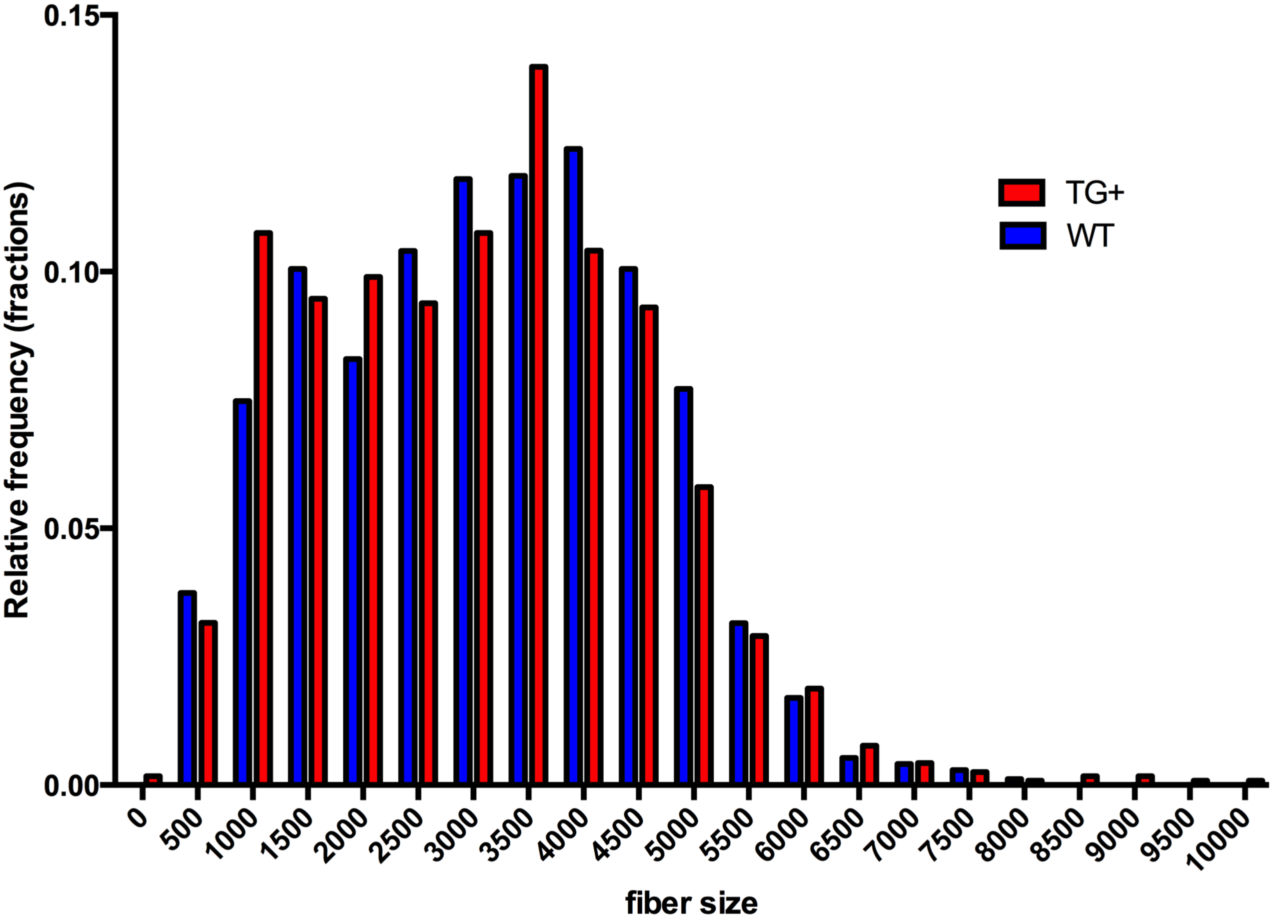

Supplement: S3 Fig — (PDF) [file pgen.1006019.s003.pdf]
