# Supplementary material for: Overexpression of Latent TGFβ Binding Protein 4 in Muscle Ameliorates Muscular Dystrophy through Myostatin and TGFβ
Source: PLoS Genet. 2016 May 5;12(5):e1006019. doi: 10.1371/journal.pgen.1006019 (PMC4858180; doi:10.1371/journal.pgen.1006019)
Supplement: S7 Fig — (PDF) [file pgen.1006019.s007.pdf]

S7 Fig. LTBP4 binding of GDF11.

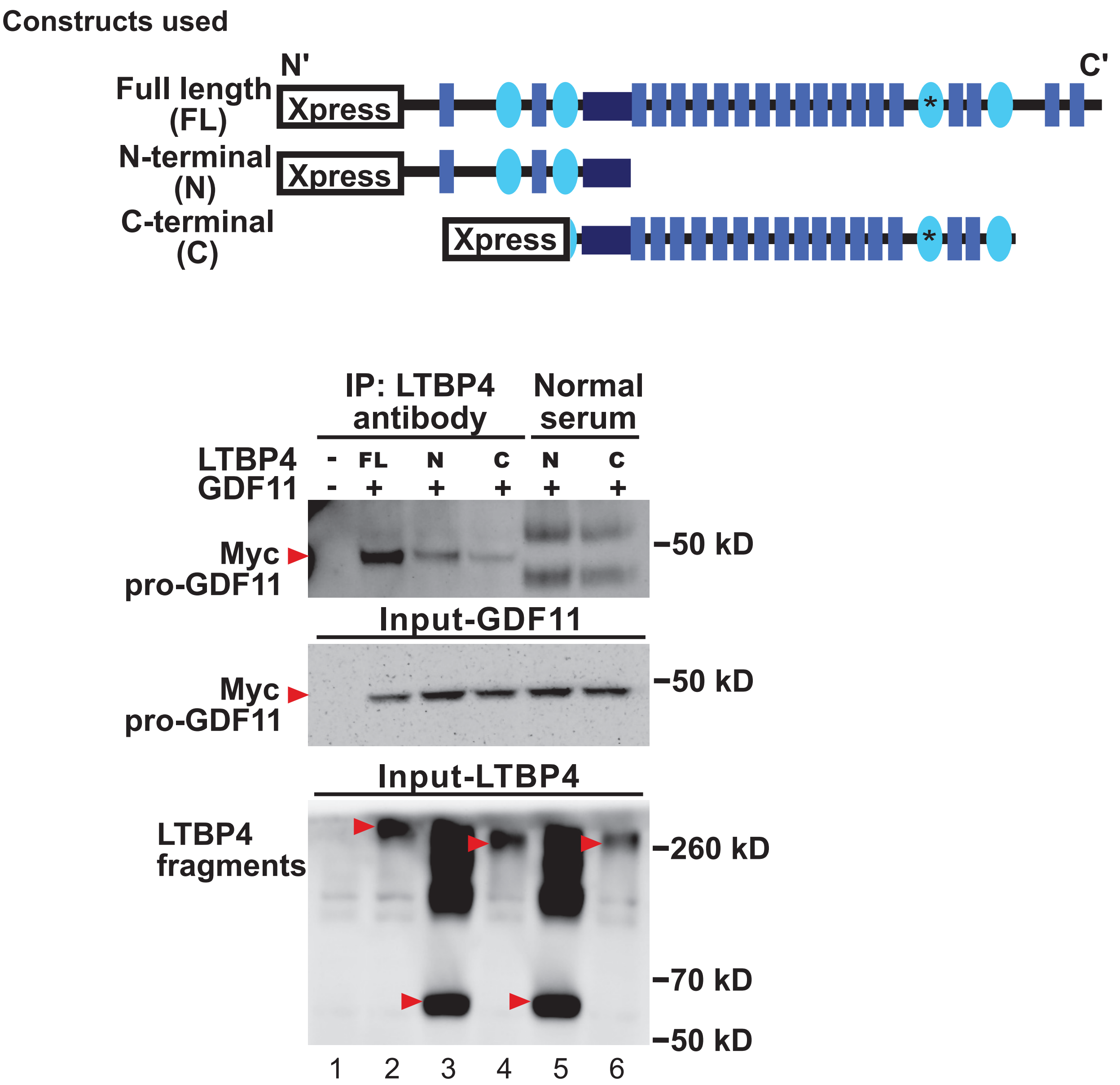

Full length (FL), N-terminal (N), or C-terminal (C) LTBP4 constructs were expressed in HEK293T cells with GDF11. FL-LTBP4 associates with GDF11 by IP (top gel panel). Both the N- and C-terminal fragments of LTBP4 associated with GDF11. The lower two panels verify that GDF11 LTBP4 were expressed as expected.
