# Supplementary material for: Overexpression of Latent TGFβ Binding Protein 4 in Muscle Ameliorates Muscular Dystrophy through Myostatin and TGFβ
Source: PLoS Genet. 2016 May 5;12(5):e1006019. doi: 10.1371/journal.pgen.1006019 (PMC4858180; doi:10.1371/journal.pgen.1006019)

S11 Fig. Schematic of constructs showing epitope tags.

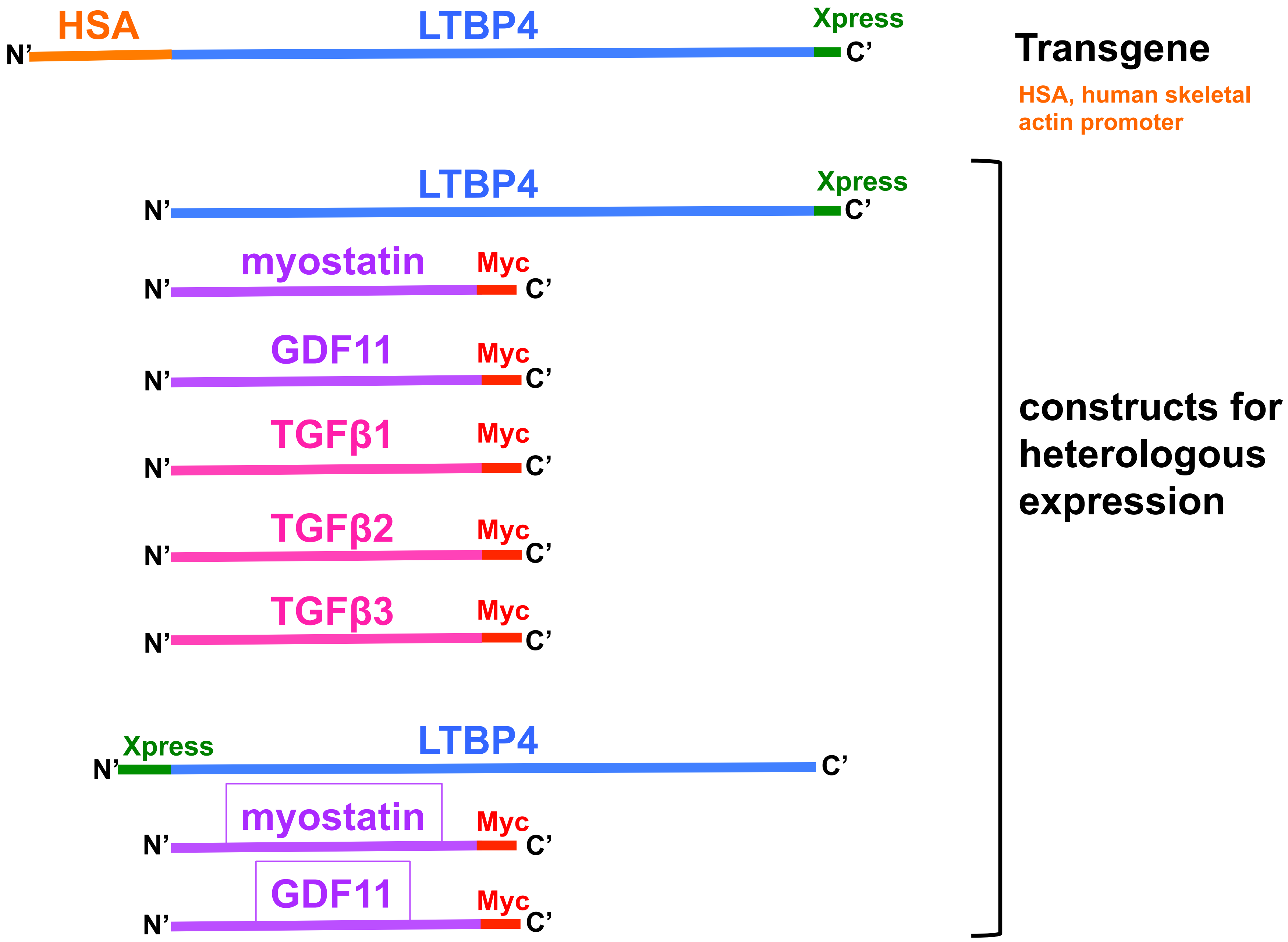

Supplement: S11 Fig — (PDF) [file pgen.1006019.s011.pdf]
